# Engineering cell sensing and responses using a GPCR-coupled CRISPR-Cas system

**DOI:** 10.1038/s41467-017-02075-1

**Published:** 2017-12-20

**Authors:** Nathan H. Kipniss, P. C. Dave P. Dingal, Timothy R. Abbott, Yuchen Gao, Haifeng Wang, Antonia A. Dominguez, Louai Labanieh, Lei S. Qi

**Affiliations:** 1 Department of Bioengineering, Stanford University, Stanford, CA 94305 USA; 2 Department of Chemical and Systems Biology, Stanford University, Stanford, CA 94305 USA; 3 Stanford ChEM-H, Stanford University, Stanford, CA 94305 USA; 4 Cancer Biology Program, Stanford University, Stanford, CA 94305 USA

## Abstract

G-protein-coupled receptors (GPCRs) are the largest and most diverse group of membrane receptors in eukaryotes and detect a wide array of cues in the human body. Here we describe a molecular device that couples CRISPR-dCas9 genome regulation to diverse natural and synthetic extracellular signals via GPCRs. We generate alternative architectures for fusing CRISPR to GPCRs utilizing the previously reported design, Tango, and our design, ChaCha. Mathematical modeling suggests that for the CRISPR ChaCha design, multiple dCas9 molecules can be released across the lifetime of a GPCR. The CRISPR ChaCha is dose-dependent, reversible, and can activate multiple endogenous genes simultaneously in response to extracellular ligands. We adopt the design to diverse GPCRs that sense a broad spectrum of ligands, including synthetic compounds, chemokines, mitogens, fatty acids, and hormones. This toolkit of CRISPR-coupled GPCRs provides a modular platform for rewiring diverse ligand sensing to targeted genome regulation for engineering cellular functions.

## Abstract (web summary)

G-protein-coupled receptors are a large and diverse group of eukaryotic membrane receptors. Here the authors couple GPCRs to dCas9 to link extracellular sensing to genome regulation.

## Introduction

Eukaryotic cells have evolved diverse classes of transmembrane receptors to transduce various extracellular signals into intracellular responses. Binding of receptors and their cognate ligands triggers their intracellular enzymatic activities. Inside the cell, this leads to complex downstream signaling protein activity and secondary messenger functions that ultimately transduce signals to genomic programs. The complexity of these signaling cascades has made it challenging to harness natural signaling pathways for cell engineering in a flexible and programmable manner^1^.

G-coupled protein receptors (GPCRs) are attractive candidates for receptor and cell engineering because they can sense a diverse repertoire of ligands, including endogenous hormones, growth factors, and natural or synthetic small molecules^2–6^. At least 800 GPCRs have been identified from the human genome and are important for regulating human physiology. Consequently, many GPCRs are implicated in diseases and are the targets of 30–40% of modern drugs^4^. Previous work in GPCR engineering replaced the intracellular domains with a proteolytically cleavable artificial transcription factor (e.g., GAL4, rtTA) to create a genetic reporter of receptor activity, called the Tango system^7, 8^. However, the use of artificial transcription factors restricted the system’s utility to exogenous genes and was unable to regulate endogenous targets. Furthermore, the system behaves as “one ligand in, one transcription factor out”, potentially limiting its efficiency. In this work, we focus on harnessing GPCRs as a generic synthetic device that can regulate the expression of endogenous genes in response to their diverse ligands.

CRISPR-Cas9 technologies have revolutionized programmable genome manipulation. Cas9 can be precisely targeted to a genomic locus of interest for gene editing using a customized single guide RNA (sgRNA)^9–11^. Beyond editing, the nuclease-deactivated Cas9 (dCas9) molecule can be fused with effector protein domains to regulate transcription of target genes or to modify the epigenome^12–18^. In addition, multiple engineered sgRNAs can be used to simultaneously regulate multiple genes and drive complex gene expression programs in response to certain ligands^14, 15, 19–22^. The flexibility of CRISPR–dCas9 systems for genome regulation makes them powerful tools for transducing receptor signaling directly to genomic targets and for programming novel sensor-coupled cell behaviors.

Here we repurpose GPCRs to convert extracellular signal sensing into programmed transcriptional responses via CRISPR-dCas9. Using an evolved GPCR that recognizes a synthetic ligand^5^, we compare two architectures for CRISPR-dCas9 gene regulation. Our design, named CRISPR ChaCha, outperforms a CRISPR Tango design^7, 8^. We formulate a simple mathematical model supported by experimental data to suggest a “one ligand in, multiple effectors out” feature of the CRISPR ChaCha system. We demonstrate that the CRISPR ChaCha system can activate individual or multiple endogenous genes targeted by dCas9/sgRNA in response to GPCR ligands, and such regulation is dose-dependent and reversible. Importantly, we devise a CRISPR ChaCha toolkit consisting of 8 GPCR–CRISPR systems that sense various synthetic compounds, chemokines, mitogens, fatty acids, and hormones. The CRISPR ChaCha toolkit presented here serves as a useful platform for understanding receptor signaling and for engineering cell-based therapies.

## Results

### Implementation of two GPCR-coupled CRISPR designs

**Fig. 1:**
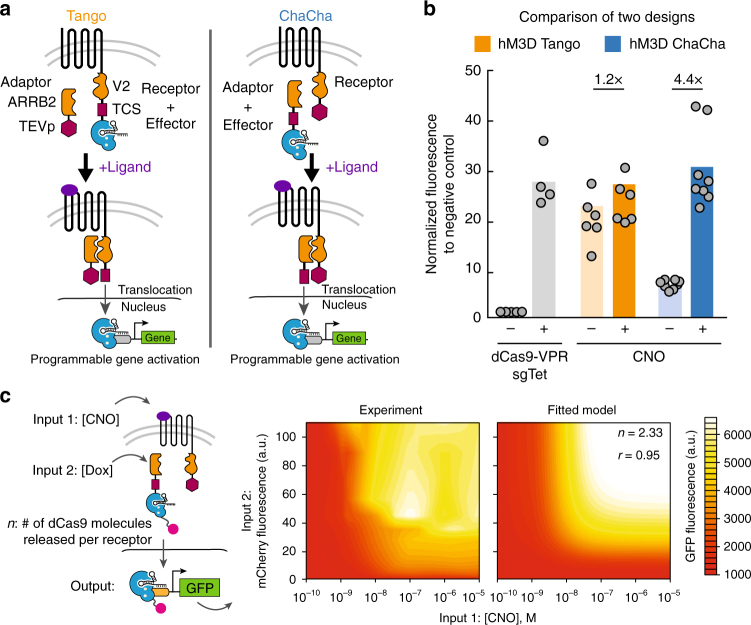
The GPCR ChaCha design outperforms the Tango design. **a** Two design schemes of coupling CRISPR-Cas9 function to the activity of GPCR. Left: the Tango design fused the effector protein (dCas9-VPR as shown) to the C-terminus of GPCR via a V2 tail sequence and a TEV cleavage sequence (TCS). An adaptor protein, Beta-Arrestin 2 (ARRB2), is fused to the Tobacco Etch Virus protease (TEVp). Right: in the ChaCha design, we fused the effector to the adaptor protein via the TCS, and fused the TEVp to the receptor via the V2 tail. Upon ligand binding to the receptor, both systems recruit the adaptor to the V2 tail, and the protease specifically cleaves at the TCS, releasing the effector protein that translocates into the nucleus for sgRNA-directed gene regulation. **b** Comparison of the performance of Tango (orange) and ChaCha (blue) designs using the synthetically evolved GPCR, hM3D in HEK293T cells with or without 20 μM clozapine-N-oxide (CNO), the ligand for hM3D (see Methods section for detailed experimental procedure). The free dCas9-VPR (gray) with and without a targeting sgRNA (sgTet) are used as positive and negative controls. The data were normalized to the free dCas9-VPR without a targeting sgRNA. The data represent two independent experiments with 4–8 technical replicates, and the bars represent the mean. **c** Estimation of the number of dCas9 molecules (*n*) released per receptor in the ChaCha design. Left, schematic of a stable cell line containing hM3D-CRISPR ChaCha with Doxycycline (Dox), inducing the expression of the ARRB2-TCS-dCas9-VPR and CNO inducing the receptor activity. Middle, GFP activation levels after 3 days of Dox induction and CNO treatment. Right, GFP activation levels based on the rate-model fit at steady state (*r* = 0.95); *n* is a measure of dCas9 molecules released per receptor (see Methods section for the detailed model)

We created and tested two designs for proteolytically coupling dCas9 function to GPCRs (Fig. 1a and Supplementary Fig. 1). The first design is derived from a previously reported Tango system^7, 8^, where the C-terminus of a GPCR is fused with a V2 tail sequence from arginine vasopressin receptor 2 (AVPR2) and typically a transcription factor (e.g, tTa). Instead, we replaced this transcription factor with a dCas9 effector (Fig. 1a, left) to create CRISPR Tango. An adaptor protein, Beta-Arrestin-2 (ARRB2), which interacts with V2 upon GPCR activation, is fused to a Tobacco Etch Virus protease (TEVp). To release the dCas9 effector, TEVp specifically cleaves the TEV cleavage sequence (TCS) placed at the N-terminus of dCas9. In the second design, we implemented a configuration, where the dCas9 effector is instead fused at the C-terminus of the ARRB2 adaptor via the TCS, and the TEVp is fused at the C-terminus of GPCR via the V2 tail (Fig. 1a, right). In this case, ligand-activated GPCR-V2-TEVp proteolytically cleaves ARRB2-TCS-dCas9 to release the dCas9 effector. After cleavage, the dCas9-effector then translocates into the nucleus to modulate genes of interest. We called this second system ChaCha, in contrast to the Tango design^7, 8^, as both represent a functional interaction between two molecules.

### The CRISPR ChaCha outperforms the CRISPR Tango

Both ChaCha and Tango designs were implemented with the evolved human muscarinic 3 GPCR (hM3-DREADD or hM3D), which recognizes the synthetic small molecule, clozapine-N-oxide (CNO)^5^. For gene activation, we used the *Streptococcus pyogenes* dCas9 fused to a tripartite transcriptional activator composed of VP64, p65 activation domain, and Rta (dCas9-VPR)^23^. We co-transfected these constructs and a TRE3G targeting sgRNA into HEK293T reporter cells, harboring a genomically integrated TRE3G promoter driving a GFP reporter (Fig. 1a and Supplementary Fig. 1; see Supplementary Table 2 for sgRNA sequences). We found that the hM3D-CRISPR ChaCha exhibited better reporter gene activation (4.4 fold) compared to the hM3D-CRISPR Tango (1.2 fold) after 1-day treatment with CNO (Fig. 1b). The Tango design displayed higher leakiness than the ChaCha design with significant GFP expression without CNO treatment. The observation that ChaCha outperforms Tango may suggest that fusing the small TEVp (28 kDa) to the receptor likely preserves the conformational fidelity and activity of the GPCR molecule, while fusing a larger domain such as dCas9-VPR (220 kDa) to GPCR may compromise its conformation by exposing the C-terminal tail of a GPCR and permit leaky interactions with ARRB2.

We applied mathematical modeling to the ChaCha architecture to characterize system behavior. Intuitively, by fusing the TEVp to the receptor, we avoid the generation of a “dead” receptor pool that can no longer be used to catalyze the release of dCas9-VPR effector. Thus, there is a potential for the ChaCha to release multiple effector across the lifetime of a receptor. To test this, we investigated the number of released dCas9-VPR molecules (defined as *n* in Fig. 1c) via experiments and mathematical modeling (see Methods section for experimental procedure and model derivation, Supplementary Data 1–4 for raw data, and Supplementary Data 5 for R scripts). We formulated a set of simple rate equations to model the effects of ARRB2-TCS-dCas9-VPR-mCherry levels and CNO concentration on target gene (GFP) activation^24^. Using a stable HEK293T cell line containing a Doxycycline (Dox)-inducible dCas9-VPR-mCherry that activated UAS promoter-driven GFP expression, we first verified that dCas9-VPR activation of GFP was not cooperative (Hill coefficient < = 1, Supplementary Fig. 2a & c). We generated another stable HEK293T reporter cell line containing the ChaCha design with a Dox-inducible ARRB2-TCS-dCas9-VPR-mCherry and a constitutively expressed receptor (Methods section), which released free dCas9-VPR molecules to activated the UAS promoter-driven GFP reporter (Fig. 1c and Supplementary Fig. 2b). Our experimental data and modeling showed GFP expression was a hyperbolic function of ARRB2-TCS-dCas9-VPR-mCherry and CNO concentration (Fig. 1c, Methods section). An unbiased, nonlinear regression fitting of GFP expression in response to varying levels of ARRB2-TCS-dCas9-VPR and CNO (Fig. 1c) revealed that the number of released dCas9 molecules per receptor (*n*) was 2.33 (*r* = 0.95), implying that multiple dCas9 molecules are released during the lifetime of a receptor.

### Design parameters that impact CRISPR ChaCha performance

We characterized various design parameters that modulate the efficiency of CRISPR ChaCha, using hM3D as a model system. These parameters included the following: (1) the linker length between ARRB2, TCS, and dCas9; (2) the proteolytic cleavage efficiency of different TCS sequences^25^; and (3) the expression of hM3D-V2-TEVp via different promoters (Supplementary Fig. 3).

Among the three parameters, our data suggested that proper placement of a flexible linker between ARRB2, TCS, and dCas9 is essential for a robust ChaCha design. More importantly, the proteolytic cleavage efficiency affects gene activation (Supplementary Fig. 3a). Somewhat surprisingly, we observed that a weaker proteolytic activity achieved the highest dynamic range of the reporter gene (Supplementary Fig. 3b). More efficient proteolytic cleavage dramatically increased the basal activation of the reporter gene in the absence of CNO, and reduced the dynamic range of activation (~2-fold). Furthermore, the receptor (hM3D-V2-TEVp) expression level affected gene activation by using different promoters (Supplementary Fig. 3c). Quantification of the expression levels of the adaptor and the receptor with different promoters suggested that a higher stoichiometry ratio of adaptor to receptor increased the dynamic range for gene activation (Supplementary Fig. 3c, d). As a result, proper choice of promoters to modulate stoichiometry between receptor and adaptor is an important consideration for optimizing the performance of GPCR-CRISPR ChaCha for a given cell type.

### Kinetics and dose response of the CRISPR ChaCha system

**Fig. 2:**
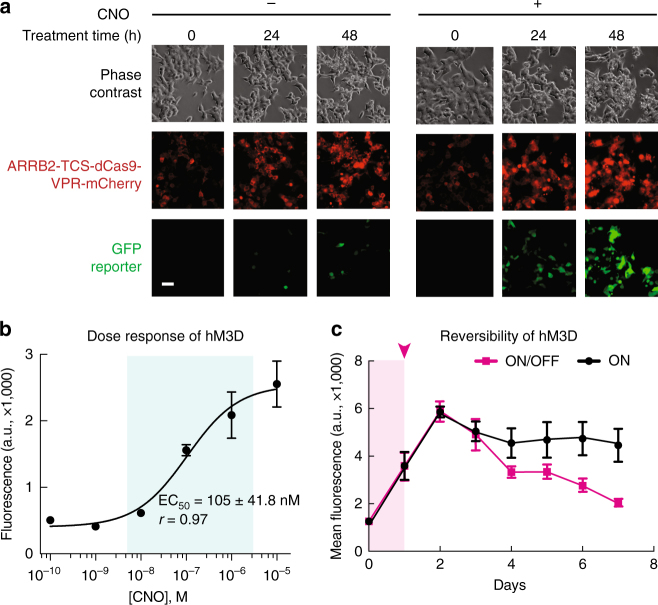
Characterization of kinetics and dose response of the CRISPR ChaCha system. **a** Time-lapse imaging of stable HEK293T cells containing hM3D-CRISPR ChaCha (see Supplementary Fig. 1 for plasmid architecture) over 48 h with or without CNO treatment. Scale bar, 50 μm. **b** The dose–response curve of hM3D-CRISPR ChaCha in HEK293T cells after 1-day treatment of different CNO concentrations. EC_50_, the effective ligand concentration to achieve half-maximal GFP induction, and is shown as mean ± standard deviation of three technical replicates. The biological replicate experimental data is shown in Supplementary Fig. 5. **c** Reversibility of the hM3D-CRISPR ChaCha system. 10 μM CNO was added to cells for 1 day (shaded magenta area) and removed after day 1 (magenta curve). As a positive control, cells were grown with CNO for 7 days (black). Each data point represents the mean of GFP fluorescence of six technical replicates from two independent experiments, and the error bars represent the standard error of the mean. See Methods section for detailed experimental procedure

To characterize the kinetics of gene regulation in the ChaCha system, we tracked GFP activation via live-cell time-lapse microscopy (Fig. 2a, Supplementary Fig. 4, and Supplementary Movies 1–3). We transfected the SFFV promoter-driven ChaCha variant into cells and treated with CNO after 24 h. We saw that mCherry-tagged ARRB2-TCS-dCas9-VPR proteins were predominantly localized to the cytoplasm during 48 h of imaging with or without CNO treatment (Supplementary Movies 1 and 2). GFP reporter expression was evident as early as 12 h after addition of CNO (Supplementary Movies 1 and 3). In contrast, samples without CNO treatment showed little GFP activation. The observation that gene activation occurs within 12 to 24 h suggests that the ChaCha system can be used to generate relatively fast cell responses to environmental stimuli.

We next investigated how gene activation by the hM3D-CRISPR ChaCha varied with CNO ligand dose using the SFFV-driven ChaCha variant (Supplementary Fig. 3c). We observed a relatively linear dose–response curve for hM3D-CRISPR ChaCha, spanning over 2 orders of magnitude of ligand induction (Fig. 2b, Supplementary Fig. 5). The fitted dose–response curve based on the Hill Equation revealed the effective CNO concentration to achieve half-maximal GFP level (EC_50_) at 105 ± 4 nM (*r* = 0.97, Fig. 2b), which is comparable to previously reported EC_50_ values using alternative methods^5^.

We further studied if the hM3D-CRISPR ChaCha is reversible upon removal of the CNO ligand. To do this, we treated the stable HEK293T reporter cell line (Supplementary Fig. 2b) with 1 μg/mL Dox for 7 days to reach steady-state expression of ARRB2-TCS-dCas9-VPR. We then treated cells with 10 μM CNO for 1 day while maintaining Dox and measured GFP expression every day for 7 days. We observed that GFP expression continued to rise for two days (due to existing free dCas9-VPR) after stopping CNO treatment and subsequently decreased to the basal level after another 3 days. In contrast, cells with sustained CNO treatment maintained GFP expression throughout the measurement (Fig. 2c). This observation that the GPCR-CRISPR ChaCha system is reversible is consistent with reports that inducible CRISPR-dCas9 gene regulation is reversible^26^.

### CRISPR ChaCha efficiently activates endogenous genes

**Fig. 3:**
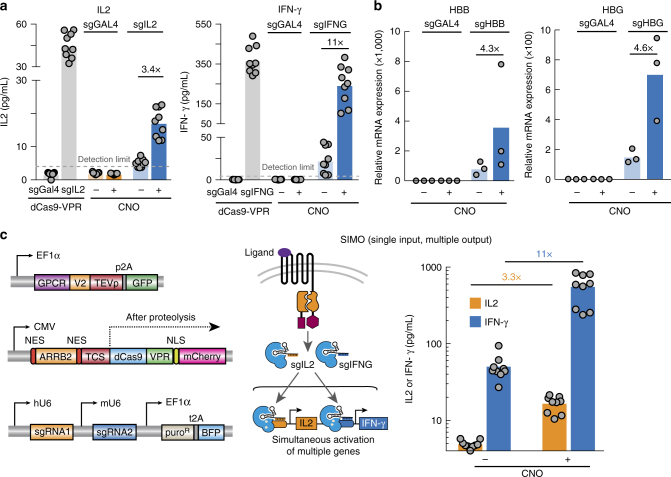
The ChaCha system enables activation of individual and of multiple genes. **a** Induction of endogenous IL2 and IFN-γ expression and secretion using the hM3D-CRISPR ChaCha system measured by ELISA. Gray, activation using free dCas9-VPR with a targeting (sgIL2 or sjpgNG) or non-targeting sgRNA (sgGAL4). Blue, activation of IL2 or IFN-γ using a targeting sgRNA (sgIL2 for IL2, sJPEGNG for IFN-γ) with or without 10 μM CNO; orange, activation using a non-targeting sgRNA with or without 10 μM CNO. The dotted line represents the detection limit of ELISA (4 pg/mL). **b** Activation of HBB and HBG using the hM3D-CRISPR ChaCha system measured by quantitative PCR (qPCR) with or without 10 μM CNO. **c** Simultaneous activation of two endogenous genes using the hM3D-CRISPR ChaCha system. Left, the plasmids of ChaCha system used for multiplexed gene regulation. Middle, a schematic overview of the ChaCha SIMO (single input, multiple output) system. Right, ELISA measure of both endogenous IL2 (orange) and IFN-γ (blue) expression and secretion using the ChaCha SIMO. For ELISA measurements, bars are the mean of three independent experiments with 7–9 technical replicates. For qPCR measurements, bars are the mean of three independent experiments that contained three technical replicates, which are then measured in technical qPCR duplicate. See Methods section for detailed experimental procedure

A key advantage of coupling GPCR signaling to the CRISPR-dCas9 system is dCas9’s ability to flexibly regulate the mammalian genome in a programmable manner. To demonstrate this ability in the GPCR-CRISPR ChaCha system, we chose to activate four endogenous genes in HEK293T cells: interleukin 2 (*IL2*) and interferon gamma (*IFNG*), which are cytokines that functionally modulate the cell-killing activity of leukocytes, and beta-globin (*HBB*) and gamma-globin (*HBG*), two genes in the human β-globin locus. All four genes are not actively expressed in HEK293T cells. We designed multiple sgRNAs that targeted the promoter region of each gene using the dCas9-VPR system, and measured the effects of sgRNAs for activating *IL2* and *IFN-γ* using the Enzyme-linked immunosorbent assay (ELISA) and the effects for activating HBB and HBG using qPCR (Supplementary Fig. 6, see Methods section). For each gene, we then used the most effective sgRNA in combination with the hM3D-CRISPR ChaCha system for ligand-mediated endogenous gene activation. After 48 h of CNO treatment, we observed efficient activation of all four genes individually tested (3.4-fold for *IL2*, and 11-fold for *IFN-γ*, Fig. 3a; 4.3 fold for *HBB*, and 4.6 fold for *HBG*, Fig. 3b; Supplementary Fig. 7). These results suggest that the CRISPR ChaCha system can activate different endogenous genes in response to its ligand.

### CRISPR ChaCha activates multiple endogenous genes

We reasoned that the CRISPR ChaCha could be used to control multiple genes simultaneously, serving as a single input, multiple output (SIMO) genetic device. To investigate this, we cloned a dual sgRNA expression vector to encode two different sgRNAs (Fig. 3c, left; Methods section). We first validated activation of *IL2* and *IFN-γ* by the dual sgRNA vector as measured by ELISA. The control experiments indicated that the dual sgRNA paired with dCas9-VPR allowed activation of each gene, and each sgRNA was specific without affecting expression of the other gene (Supplementary Fig. 8a). When we transfected the dual sgRNA vector with the hM3D-CRISPR ChaCha system, we observed simultaneous activation of both cytokines in response to CNO treatment (3.3-fold for *IL2* and 11-fold fold *IFN-γ*, Fig. 3c). Compared to using a single sgRNA, we observed similar activation fold change (Fig. 3a and Supplementary Fig. 8b), suggesting the system is suitable for activating multiple genes without comprised efficiency. Together, our data confirmed that the GPCR-coupled CRISPR ChaCha system could efficiently modulate endogenous genes in response to GPCR signals in human cells.

### Generation of a CRISPR ChaCha toolkit using different GPCRs

**Fig. 4:**
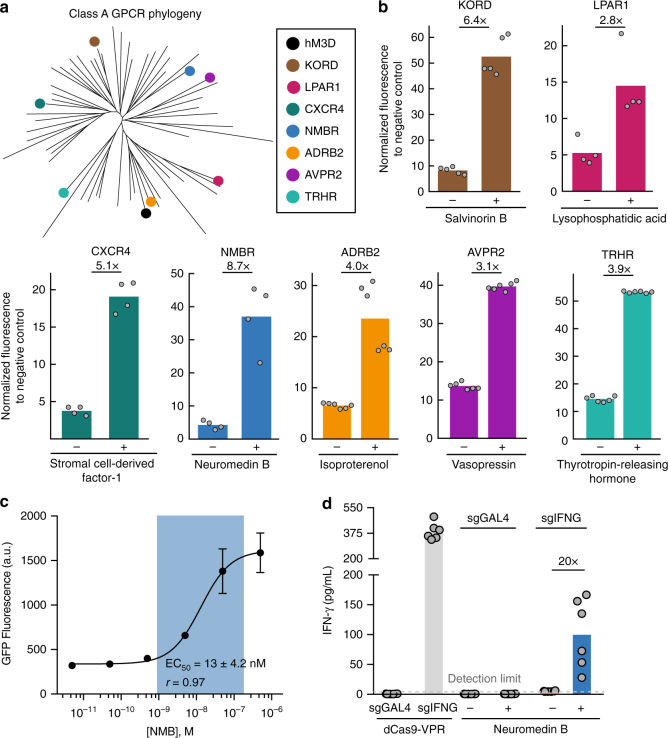
Expanding the ChaCha design to other synthetic and natural GPCRs. **a** The phylogenetic tree of Class A GPCRs. Synthetic (hM3D, KORD) and natural (LPAR, CXCR4, NMBR, ADRB2, AVPR, TRHR) GPCRs tested here are indicated. **b** The measured performance of diverse GPCRs with the ChaCha architecture for their respective ligands using HEK293T-GFP reporter cell line (see Supplementary Table 4 for ligand concentrations). The data were normalized to the free dCas9-VPR without a targeting sgRNA (for KORD, LPAR, CXCR4, NMBR) or with a non-targeting sgRNA (for ADRB2, AVPR, TRHR). The data represent two independent experiments with 2–3 technical replicates, and the bars represent the mean. For CXCR4, NMBR, and LPAR, we chose the best performing version characterized from a few variants as shown in Supplementary Fig. 9. See Methods section for the experimental procedure. **c** The dose–response curve of NMBR-CRISPR ChaCha in HEK293T cells after 1-day treatment of different CNO concentrations. EC_50_, the effective ligand concentration to achieve half-maximal GFP induction, and is shown as mean ± s.d. of three technical replicates. The replicate experimental data is shown in Supplementary Fig. 10. **d** Induction of endogenous IFN-γ by NMBR-CRISPR ChaCha in HEK293T cells after 2 days of 0.5 μM NMB treatment. sgGAL4, non-targeting sgRNA; sgIFNG, *IFNG*-targeting sgRNA.+/− indicates with or without Neuromedin B. The fold of activation displayed on top of bars compares+/− treatment conditions. The bars represent the mean, and the data represent two independent experiments with 3 technical replicates

GPCRs are the largest and most diverse group of membrane receptors in the human body, with Class A GPCRs constituting over 80% of the entire family^4, 8^. We next tested the generality of the ChaCha design for more Class A GPCRs. We chose seven additional GPCRs that were classified on different branches of the Class A GPCR phylogenetic tree (Fig. 4a). These included another evolved GPCR, the kappa opioid receptor DREADD (KORD), which senses Salvinorin B^6^ and six natural GPCRs: CXCR4, which senses the chemokine stromal derived factor 1 (SDF1); NMBR, which senses the mitogen neuromedin B; LPAR1, which senses the fatty acid lysophosphatidic acid; ADRB2, which senses the hormone epinephrine and similar compounds; AVPR2, which senses the hormone vasopressin; and TRHR, which senses thyrotropin-releasing hormone^8, 27^ (see Supplementary Table 4 for ligands used and their concentrations).

Notably, the ChaCha design worked efficiently for all seven additional GPCRs tested, with NMBR exhibiting the best activity in response to its ligand (Fig. 4b). Interestingly, we observed efficient gene activation for TRHR, which has not been reported for the Tango design^8^. We also tested different linkers for fusing TEVp to the C-terminal of a subset of these GPCRs (CXCR4, NMBR, LPAR1), and observed that the alterations in the V2 tail length and composition was important for better ON/OFF activity (Supplementary Fig. 9).

Using NMBR-CRISPR ChaCha as an example, we further measured the dose-response behavior. In contrast to the hM3D receptor, the NMBR receptor displayed a narrower induction range (Fig. 4c and Supplementary Fig. 10). The fitted dose–response curve using the Hill Equation revealed an effective NMB concentration to achieve half-maximal GFP level (EC_50_) at 13 ± 4.2 nM (*r* = 0.97, Fig. 4c), comparable to the reported EC_50_ values measured using alternative methods (27 nM^28^, or 2 nM^8^). We also tested whether a natural GPCR-CRISPR ChaCha can activate endogenous genes. Using the NMBR-CRISPR ChaCha, we observed 20-fold activation of the endogenous IFN-γ expression in HEK293T cells after 2-day NMB treatment (Fig. 4d). The toolkit of 8 CRISPR ChaCha systems reported here greatly expands the ligand-inducible control of CRISPR-dCas9 for efficient endogenous gene regulation, suggesting that the strategy should be generalizable to many other Class A GPCRs.

## Discussion

Here we established a strategy named the ChaCha design to couple programmable gene regulation function of CRISPR-dCas9 with the ligand-sensing ability of GPCRs. We demonstrated this strategy by devising a CRISPR-coupled GPCR toolkit consisting of two synthetic GPCRs and six natural GPCRs. These CRISPR ChaCha systems can modulate gene expression in response to diverse ligands including synthetic compounds (CNO, Salvinorin B, and Isoproterenol), hormones (vasopressin, thyrotropin-releasing hormone), mitogens (neuromedin B), chemokines (stromal derived factor 1), and fatty acids (lysophosphatidic acid), which greatly expands the tools for inducible control of the human genome via the CRISPR–Cas system. Together, the developed CRISPR ChaCha systems provide an efficient and modular platform that employs diverse GPCRs for programmable sensing of a broad spectrum of ligands for modulating gene expression. Given the generality of the ChaCha design, we expect this approach can be applied to more GPCRs for more ligand inputs.

The CRISPR-coupled GPCR ChaCha system presents several advantages. First, fusing the small TEVp to the receptor likely preserves the conformational fidelity and activity of the GPCR molecule. Indeed, our data showed that the hM3D ChaCha improved the dynamic range of gene activation compared to the hM3D Tango (Fig. 1b). Second, we used a simple rate model and experimental data regression (Methods section) to show that the ChaCha design could allow for the release of multiple dCas9 effectors (estimated to be 2.33) during the life cycle of a receptor while the Tango design can maximally release one effector per receptor. Third, the programmability of CRISPR–Cas system allows us to easily redirect the same (synthetic or natural) signal to different genomic outputs (e.g. endogenous IL2, IFN-γ, HBB, and HBG). This greatly expands previous approaches beyond the use of synthetic transcription factors (e.g., GAL4 or tTA) that can only produce synthetic outputs from delivered transgenes.

The ChaCha system can be further optimized to overcome a few current limitations. While the ChaCha system is less leaky than Tango (Fig. 1b), we observed some level of leakiness. We performed experiments to probe the sources of observed leakiness. Our data suggested that the basal signaling activity of receptor without the ligand and the basal cleavage of TCS by the TEVp were the main source of the leakiness (Supplementary Fig. 11). Adopting a weaker TEVp, a weaker TCS (Supplementary Fig. 3b), or an inducible TEVp may lower this leakiness. Furthermore, using promoters that optimize the stoichiometric ratio between the receptor and adaptor molecules could increase the dynamic range of gene regulation (Supplementary Fig. 3c).

The ChaCha system can benefit from diverse CRISPR tools that are being developed. For example, combining the system with diverse species of dCas9 should broaden the targetable genome space^29, 30^; coupling the system with the dCas9-mediated repressors may result in ligand-mediated repression of endogenous genes; combining with epigenetic regulators may further enable ligand-mediated epigenome editing of the mammalian genome^16, 18, 31^.

While this manuscript was under review, another work was reported that coupled the CRISPR–Cas system to GPCRs using the Tango architecture and a split dCas9 strategy^32^. In their system, the split dCas9 strategy reduced the leakiness of the system by limiting the activity of full dCas9 molecules for undesired transcription control. Our ChaCha architecture provides an alternative, complementary strategy, which could also reduce the leakiness by avoiding fusing a large dCas9 molecule to the receptor, and potentially allow for the release of multiple effectors per receptor to enhance target gene activation. We expect that a combination of both strategies should provide an approach to achieve robust, sensitive, efficient CRISPR-coupled GPCR devices in the future.

Probing GPCR activity in vivo has been a useful avenue for understanding their function^33^ and the ligands of many orphan GPCRs remain elusive^34^. The engineered ChaCha systems may help understand GPCR signaling and probe GPCR activity in response to their ligands. For example, one of the receptors tested (TRHR) was classified as “non-orphan, but also non-optimized” for transcriptional read-out by the Tango assay^8^, despite being described to interact with ARRB2^27^. Our result showed TRHR ChaCha worked well, suggesting it may be possible for a broader family of GPCRs to be adopted to transcriptional outputs with the ChaCha design. This would not only facilitate profiling GPCR signaling, but further expand diverse convertible inputs across the GPCR phylogenetic tree for dCas9 genome regulation.

We further envision that the CRISPR ChaCha system can be used to create novel cell therapeutic programs. Given the diverse ligand spectrum of GPCRs and their importance in development, physiology, and disease^2, 4^, harnessing and rewiring GPCR pathways may incorporate physiological or disease-relevant stimuli that cannot be detected using antibody-based approaches^35, 36^. For example, human primary T cells engineered with chimeric antigen receptor (CAR)^37^ or SynNotch^35^ could be equipped with CRISPR ChaCha to detect GPCR ligands in the tumor microenvironment^38^ and conditionally execute tumoricidal functions. Additionally, given the pharmacological relevance of GPCRs, the toolkit also offers an avenue for external control of the CRISPR function using synthetic compounds in vivo^5, 6, 39^.

Altogether, the CRISPR ChaCha system represents a significant methodological advancement for connecting diverse extracellular signals to the CRISPR-dCas9 gene regulation program, and is useful for both understanding receptor biology and devising cell-based therapeutics.

## Methods

### Generation of genetic constructs

Standard molecular cloning techniques were performed to assemble all constructs used in this paper and they are included in Supplementary Table 1.

Human codon-optimized *S. pyogenes* dCas9 was fused at the C-terminus with the tripartite VPR activator.^23^ VPR is a fusion of VP64, p65 activation domain, and Rta via two GS linkers. An SV40 nuclear localization signal (NLS, PKKKRKV) was inserted C-terminal to VP64. For visualization, mCherry was fused at the C-terminus of the construct. The fusion construct was cloned into a pcDNA3 vector with a CMV promoter driving the expression of dCas9-VPR-mCherry. For the mathematical model, a lentiviral pHR vector with a Doxycycline (Dox)-inducible TRE3G promoter was used instead.

ARRB2-TCS-dCas9-VPR was assembled by fusing ARRB2 (Human cDNA, NM_004313.3; Origene) with dCas9-VPR-mCherry and cloned into a pcDNA3 vector. The TCS sequence ENLYFQ/X was inserted in between and was flanked with GS linkers of varying lengths (Supplementary Fig. 3). Two nuclear export signals (NES, LALKLAGLDI) flanked ARRB2 to ensure cytoplasmic localization of the chimera. For the mathematical model, a lentiviral pHR vector with a Dox-inducible TRE3G promoter was used instead.

Synthetic GPCRs, natural GPCRs and TEV protease (Addgene #8835) were all PCR amplified and cloned into a pHR lentiviral vector by InFusion (Takara Clontech) cloning. The V2 sequence (derived from AVPR2)^7^ was inserted in between GPCR and TEVp as primer overhangs via InFusion cloning. For visualization, p2A-BFP was fused C-terminal to TEVp. Expression of GPCR-V2-TEVp-p2A-BFP was driven by an EF1a, PGK or SFFV promoter. See Supplementary Table 3 for plasmid sources for receptors.

All sgRNAs were cloned into a pHR lentiviral U6-driven expression vector that co-expressed puromycin-p2A-BFP or upstream of the GPCR-V2-TEVp locus for ease of transfection of the three-component GPCR-CRISPR ChaCha system. Alternative sgRNA sequences were generated by PCR and inserted by InFusion cloning into the vector digested with BstXI and NotI (New England Biolabs).

For multiplexing experiments, we also cloned a dual sgRNA vector to otherwise reduce false positives in bulk measurements (e.g., ELISA). This consists of two sgRNA cassettes in tandem driven by mouse U6 (mU6) and human U6 (hU6) promoters, respectively, and a co-expressed puromycin-p2A-BFP cloned into a pHR lentiviral vector. Here, the mU6 vectors are cloned using InFusion (Clontech) to insert PCR products into a modified vector digested with BstXI and SpeI. The hU6 sgRNA vector was cloned inserting PCR productions with InFusion cloning into a parent vector digested with XbaI and SpeI. After sequence verification, vectors were combined by digesting the XU6 sgRNA with XbaI and SalI, taking the insert and ligating into a SpeI and SalI digested XU6 vector.

Below is the standard *S*. *pyogenes* sgRNA scaffold used (N’s denote the spacer sequence):

5′-NNNNNNNNNNNNNNNNNNNNGTTTAAGAGCTATGCTGGAAACAGCATAGCAAGTTTAAA TAAGGCTAGTCCGTTATCAACTTGAAAAAGTGGCACCGAGTCGGTGCTTTTTTT-3′.

Spacer sequences for all sgRNAs used can be found in Supplementary Table 2.

### Cell culture and generation of stable cell lines

HEK293T cells (Lenti-X^TM^, Clontech) were maintained in DMEM High Glucose with GlutaMAX^TM^ media (Thermo Fisher) supplemented with 10% Tet System Approved FBS (Clontech) and 100 U/mL of penicillin and streptomycin (Gibco) at 37 ^o^C with 5% CO_2_. We did not independently authenticate these cell lines and they were not tested for mycoplasma contamination.

For transfection, HEK293T cells (Lenti-X^TM^, Clontech) with 3 μL of Mirus TransIT-LT1 reagent per μg of plasmid added, and then incubated at room temperature for 15–30 min. Unless otherwise noted, GPCR ligands were added at the following concentrations at day 3 as specified in Supplementary Table 4. This table also specifies the media conditions used for each receptor to generate the data in Fig. 4.

We used lentiviral transduction to generate stable cell lines. At day 1, cells were seeded at 2.0–3.0 × 10^5^ cells/mL in a 6-well plate format (Corning). At day 2, cells were 50–70% confluent at the time of transfection. For each well, 1.51 μg of pHR vector containing the construct of interest, 1.32 μg of dR8.91 and 165 ng of pMD2.G were mixed in 250 μL of Opti-MEM reduced serum media (Gibco) with 7.5 μL of Mirus TransIT-LT1 reagent and incubated at room temperature for 15–30 min. The transfection complex solution was distributed evenly to HEK293T cultures dropwise. Media was replaced at day 3 with fresh media. At day 4, lentiviruses are harvested from the supernatant with a sterile syringe and filtered through a 0.45-μm polyvinylidene fluoride filter (Millipore) for immediate transduction of target cell cultures.

Filtered lentiviral supernatants were mixed 1:1 with appropriate fresh media to replace media of target cells for transduction. Adherent cell cultures were transduced at 50% confluence. Approximately 10 days after transduction, the HEK293T pTRE3G-GFP line and the pUAS-GFP::pEF1α-rtTA-p2A-puro reporter line (pre-selected for 2 days with 1 μg/μL puromycin) were transiently transfected with dCas9-VPR and a targeting sgRNA (sgTET and sgUAS, respectively) for 1 day prior to sorting via GFP FACS in Carmen (BD InFlux) and Aida (BD Aria II) sorters, respectively. For the rate model, the HEK293T pUAS-GFP::pEF1a-rtTA-p2A-puro line was transduced with pEF1α-hM3D-V2-TEVp-p2A-BFP and pTRE3G-(ARRB2-TCS)-dCas9-VPR-mCherry and sorted ~7 days after transduction for both BFP and 1-day doxycycline induction of mCherry expression.

### Flow cytometry analysis

Cell were dissociated using 0.05% Trypsin-EDTA (Life Technologies) and analyzed for reporter fluorescence in the Stanford Shared FACS facility with a Scanford FACScan analyzer (Becton Dickinson), or with a CytoFLEX S flow cytometer (Beckman Coulter). We collected 10,000 cells containing constructs of interest for analysis (BFP and mCherry double positive). The data presented are normalized to either a free dCas9-VPR with no sgRNA, or non-targeting sgRNA control as specified.

### Time-lapse microscopy

At day 0, HEK293T TRE3G-GFP reporter cells were plated at 1 × 10^5^ cells per 24-well well (μ-Plate 24 well; ibidi). At day 1, 250 ng of each plasmid was transfected (see Fig. 2a and Supplementary Fig. 4). At day 2, 20 μM of CNO was added to appropriate wells and immediately imaged. Time-lapse microscopy was performed on a Leica DMi8 inverted microscope equipped with, Lumencor SOLA SMII 405, Leica DFC9000 GT camera and Oko-Lab cage incubation system at 37 ^o^C with 5% CO_2_. Leica Application Software was used to set up time-lapse imaging. Images from phase contrast, mCherry (filter cube TXR, No. 11525310), and GFP (filter cube GFP, Cat. No. 11525314) channels were taken every 0.5 h for 48 h with a 20x/0.40NA corr PH1 objective using Leica Adaptive Focus control. Image processing was performed in Fiji (ImageJ).

### Reversibility experiment

A stable HEK293T line containing pUAS-GFP, pEF1a-rtTA-p2A-puro, pEF1α-hM3D-V2-TEVp-p2A-BFP, and pTRE3G-(ARRB2-TCS)-dCas9-VPR-mCherry (Supplementary Fig. 2b) was pre-induced with 1 μg/mL Dox for seven days to stabilize ARRB2-TCS-dCas9-VPR levels. Cells were then treated with 10 μM CNO either for one to seven days, or for 1 day and removed for 1–6 days. All cells were measured by flow cytometry on the same day, collecting 10,000 mCherry and BFP double positive cells for analysis.

### Endogenous cytokine activation and secretion assays

A day before transfection, HEK293T cells were seeded in 24-well plates at a density of 5 × 10^4^ cells per well. On day 1, cells were transfected with 250 ng of each plasmid (i.e., the CRISPR ChaCha components: GPCR-V2-TEVp of interest, the ARRB2-TCS-dCas9-VPR, and an sgRNA). On day 2, controls were transfected, consisting of the GPCR of interest, dCas9-VPR, and an sgRNA. Media on the ChaCha-containing cells was then changed to those with or without ligand treatment (10 μM for CNO; 0.5 μM for NMB).

Supernatants from cell cultures were harvested on day 4, and stored at −80 ^o^C. Secreted proteins were quantified using the ELISA MAX Deluxe kits for human IL2 and IFN-γ (BioLegend). Absorbance at 450 nm and 570 nm was measured for samples in technical triplicates with a Synergy H1 plate reader (BioTek). Samples were standardized by subtracting measurements at 570 nm from those at 450 nm. Protein concentrations were then determined by standard curves fitted to a power law using Excel (Microsoft).

### qPCR analysis of gene expression

Cells were transfected as described in the proceeding section. On day 4, cells were harvested and RNA was extracted using a RNeasy Midi Plus Kit (Qiagen). cDNA was then prepared using 500 ng of RNA per 20 μL reaction via iSCRIPT cDNA synthesis (BioRad). Following synthesis, cDNA was stored at −30 °C until qPCR.

qPCR was conducted in 10 μL reactions using 384 well plates, using 15 ng of cDNA, a 400 nM final concentration of primers, and iTaq Universal SYBR Green Supermix (BioRad). See Supplementary Table 5 for primers used. From transfection, there were three technical triplicates that were then ran in technical duplicate for qPCR reactions. Thermocycling was done as follows: 95° for 1 min, 95° for 10 s, and 60° for 30 s. The latter two steps cycled for 50 repeats with plate reads taken after the 60° step^40^ on a CFX384 Touch Real-Time PCR thermocycler (BioRad). To reduce technical variation in loading 384 well plates, each independent experiment was ran on the same day with the same aliquots of a qPCR reaction master mix. We applied a Ct threshold of 35 cycles after running water controls for each primer. Thus, any Ct values that were over 35 or not reported after 50 cycles were then set to a Ct of 35 cycles.

The data were analyzed using the ΔΔ*C*_t_ method. Δ*C*_t_ was calculated about the housekeeping gene GAPDH. Then ΔΔ*C*_t_ was calculated using the Δ*C*_t_ t from the gene of interest (GOI), subtracted from the Δ*C*_t_ of the free dCas9-VPR and sgGal4 condition (*M*_*0*_). We then report relative expression as the following:

$${\mathrm{Relative}}\,{\mathrm{expression}} = 2^{ - \left( {\left( {C_{\mathrm{t}}^{{\mathrm{GOI}}} - C_{\mathrm{t}}^{{\mathrm{GAPDH}}}} \right) - \left( {C_{\mathrm{t}}^{M_0} - C_{\mathrm{t}}^{{\mathrm{GAPDH}}}} \right)} \right)}$$

Fold changes are reported as the ratio of relative expression between the CNO treated and untreated conditions.

### Class A GPCR phylogenetic tree construction

The phylogenetic tree in Fig. 4a was constructed using GPCRdb^41, 42^. Human GPCRs from the Swiss-Prot database were used as reference, without any selection for G protein preference. One GPCR from each family of Class A/Rhodopsin Family GPCRs was used to construct the tree, including those utilized in this study. Full-length sequences of receptors were considered for tree construction. No bootstrapping was performed, and distance calculation utilized the neighbor-joining method, with the regular branch lengths option. The tree was then rendered using T-REX^43^.

### Modeling GFP activation by doxycycline-inducible dCas9-VPR

We construct rate equations to model the induction of dCas9-VPR-mCherry (referred hereafter simply as dCas9) by Dox (*D*) and the dCas9-induced activation of the target reporter gene, GFP.

1 $$\frac{{{\mathrm{d}}C}}{{{\mathrm{d}}t}} = \alpha _{\mathrm{1}} \cdot \frac{{D^n}}{{K_{\mathrm{D}} + D^n}} - \beta _{\mathrm{1}} \cdot C$$

2 $$\frac{{{\mathrm{d}}G}}{{{\mathrm{d}}t}} = \alpha _{\mathrm{2}} \cdot C^m - \beta _{\mathrm{2}} \cdot G$$

where *α*_1_ and *α*_2_ are first-order rate constants for dox-induced dCas9 (*C*) production and subsequent dCas9-induced production of GFP (*G*), respectively; the Hill coefficient *n* and *K*_*D*_ are the cooperativity and affinity constants of dox induction, respectively; the exponent *m* is a lumped parameter that captures the following processes in series: dCas9 binding to the gene target (GFP), transcription, and translation of GFP; *β*_1_ and *β*_2_ are first-order degradation rate constants for dCas9 and GFP, respectively.

At steady state,

3 $$\frac{{{\mathrm{d}}C}}{{{\mathrm{d}}t}} = \frac{{{\mathrm{d}}G}}{{{\mathrm{d}}t}} = 0$$

which yields steady state (ss) formulae for *C* and *G*

4 $$C_{{\mathrm{ss}}} = \kappa _{\mathrm{1}} \cdot \frac{{D^n}}{{K_{D} + D^n}}$$

5 $$G_{{\mathrm{ss}}} = \kappa _{\mathrm{2}} \cdot C_{{\mathrm{ss}}}$$

6 $$G_{{\mathrm{ss}}} = \kappa _{\mathrm{1}} \kappa _{\mathrm{2}} \cdot \frac{{D^n}}{{K_{D} + D^n}}$$

where *κ*_1_ = *α*_1_ */β*_1_, *κ*_2_ = *α*_2_ */β*_2_, and *G*_max_ = *κ*_1_ *κ*_2_; *G*_max_ represents the theoretical maximum GFP level.

### A simple mathematical rate model of the CRISPR ChaCha system

We construct rate equations to model four connected processes, which are: (i) conversion of inactive hM3D-TEV (*R*) receptor to an activated state (*R* ^*^) upon CNO ligand (*L*) binding, which leads to (ii) the cleavage of dCas9-VPR-mCherry (*C*) from ARRB2-dCas9-VPR-mCherry (*A*, referred hereafter simply as ARRB2-dCas9) that can be (iii) induced with doxycycline (*D*), and (iv) the subsequent activation of the target reporter gene, GFP (*G*), by cleaved dCas9-VPR.

7 $$\frac{{{\mathrm{d}}R}}{{{\mathrm{d}}t}} = \alpha _{\it{R}} - \alpha _{{\it{R}}^*} \cdot {\it{RL}} - \beta _{\it{R}} \cdot R$$

8 $$\frac{{{\mathrm{d}}R^{\mathrm{*}}}}{{{\mathrm{d}}t}} = \alpha _{R^{\mathrm{*}}} \cdot RL - \beta _{R^{\mathrm{*}}} \cdot R^{\mathrm{*}} - \gamma _C \cdot R^{\mathrm{*}}A^n$$

9 $$\frac{{{\mathrm{d}}A}}{{{\mathrm{d}}t}} = \alpha _A - \beta _A \cdot A - \gamma _C \cdot R^{\mathrm{*}}A^n$$

10 $$\frac{{{\mathrm{d}}C}}{{{\mathrm{d}}t}} = \gamma _C \cdot R^{\mathrm{*}}A^n - \gamma _G \cdot C - \beta _C \cdot C$$

11 $$\frac{{{\mathrm{d}}G}}{{{\mathrm{d}}t}} = \gamma _G \cdot C - \beta _G \cdot G$$

Where *α*_*R*_, *α*_R*_ and *α*_*A*_ are production rate constants for inactive receptor, ligand-activated receptor, and ARRB2-dCas9; *β*_*R*_, *β*_R*_, *β*_*A*_, *β*_*C*_, and *β*_*G*_ are first-order degradation rate constants for inactive receptor, active receptor, ARRB2-dCas9, cleaved dCas9, and GFP, respectively; γ_*C*_ and γ_*G*_ are reaction rate constants for active receptor-mediated cleavage of ARRB2-dCas9 to release dCas9, and subsequent dCas9-induced production of GFP, respectively; *n* is the number of ARRB2-dCas9-VPR molecules recruited per one active receptor.

At steady state, all time derivatives go to zero, which yield the following steady state (ss) formulae for relevant molecules

12 $$R_{{\mathrm{ss}}} = \frac{{\alpha _R}}{{\alpha _{R^{\mathrm{*}}} \cdot L + \beta _R}}$$

13 $$R^{\mathrm{*}}_{{\mathrm{ss}}} = \frac{{\alpha _{R^*} \cdot R_{{\mathrm{ss}}}L}}{{\beta _{R^{\mathrm{*}}} + \gamma _C \cdot A_{{\mathrm{ss}}}^n}}$$

14 $$C_{{\mathrm{ss}}} = \frac{{\gamma _C \cdot R^{\mathrm{*}}_{{\mathrm{ss}}}A_{{\mathrm{ss}}}^n}}{{\gamma _G + \beta _C}}$$

15 $$G_{{\mathrm{ss}}} = \frac{{\gamma _G}}{{\beta _G}} \cdot C_{{\mathrm{ss}}}$$

Substituting equations (12), (13), (14) into Equation (15) yields a steady-state formula for GFP as a function of CNO ligand and ARRB2-dCas9,

16 $$G_{{\mathrm{ss}}} = \frac{{\gamma _G}}{{\beta _G}} \cdot \frac{{\alpha _R}}{{\gamma _G + \beta _C}} \cdot \frac{L}{{L + \beta _R/\alpha _{R^{\mathrm{*}}}}} \cdot \frac{{A_{{\mathrm{ss}}}^n}}{{\beta _{R^{\mathrm{*}}}/\gamma _C + A_{{\mathrm{ss}}}^n}}$$

or simply,

17 $$G_{{\mathrm{ss}}} = G_{{\mathrm{max}}} \cdot \frac{L}{{L + K_L}} \cdot \frac{{A_{{\mathrm{ss}}}^n}}{{K_A + A_{{\mathrm{ss}}}^n}}$$

where $G_{{\mathrm{max}}} = \frac{{\gamma _G}}{{\beta _G}} \cdot \frac{{\alpha _R}}{{\gamma _G + \beta _C}}$ represents the theoretical maximum GFP level at high saturating levels of *L* and *A*, and it is a function of the rate constants for receptor production, dCas9 degradation, and GFP degradation; $K_L = \beta _R/\alpha _{R^*}$ represents the set-point concentration for the CNO ligand to produce half-maximal GFP levels; $K_A = \beta _{R^*}/\gamma _C$ represents the ratio of active receptor-mediated ARRB2-dCas9 cleavage and active receptor degradation rate constants.

### Data presentation and analyses

Data are displayed as individual points with sample size indicated in figure legends. No sample size estimates were performed, and the sample sizes used in this study are consistent with those used by similar genome editing and gene regulation studies. Experiments were performed independently at least two times. Values reported are relative to indicated control conditions. No randomization or blinding was performed.

Statistical analysis was performed using SPSS Statistics 21 (version 22, IBM Corporation), or Prism 7 (Graphpad). Equal variance between populations was not assumed. To account for unequal variance among conditions, Welch’s two-sided *t* test was performed when comparing two conditions, and Welch’s ANOVA was performed followed by Games–Howell post hoc tests when comparing more than two conditions with each other. All statistical data analyses are compiled in Supplementary Table 6.

### Data availability

All relevant data can be provided by the authors. In the manuscript we provide the raw data (Supplementary Data 1–4) and R scripts (Supplementary Data 5) used to generate in Fig. 1 and Supplementary Fig. 2.

## Electronic supplementary material

Supplementary Information

Description of Additional Supplementary Files

Supplementary Data 1

Supplementary Data 2

Supplementary Data 3

Supplementary Data 4

Supplementary Data 5

Supplementary Movie 1

Supplementary Movie 2

Supplementary Movie 3
