# Supplementary material for: Engineering cell sensing and responses using a GPCR-coupled CRISPR-Cas system
Source: Nat Commun. 2017 Dec 20;8:2212. doi: 10.1038/s41467-017-02075-1 (PMC5738360; doi:10.1038/s41467-017-02075-1)
Supplement: Supplementary file 1 — Supplementary Information [file 41467_2017_2075_MOESM1_ESM.pdf]

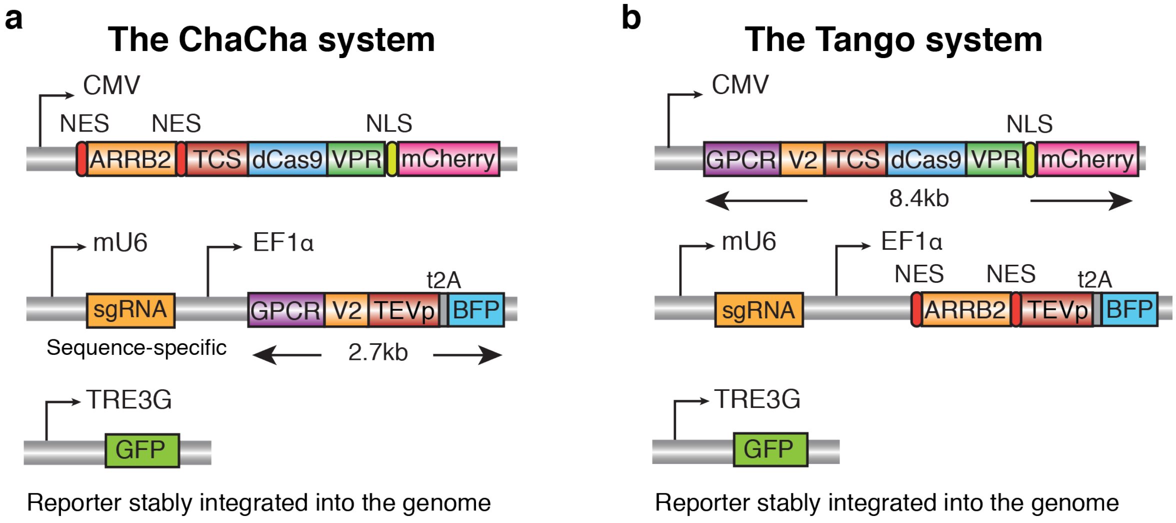

Supplementary Figure 1 | The plasmids used for the GPCR-CRISPR ChaCha and the GPCR-CRISPR Tango systems. (**a**) The plasmids for the GPCR-CRISPR ChaCha system. The dCas9-VPR effector is fused to the C-terminus of a nuclear export signal (NES)-flanked Beta-Arrestin 2 (ARRB2) with a TEV protease cleavage site (TCS), and a small linker. Embedded in the VPR activator is a nuclear localization signal (NLS), and at the C-terminus mCherry is fused for visualization. A single guide RNA (sgRNA) is expressed by the murine U6 (mU6) promoter, and a shown stream cassette contains EF1α-driven GPCR with the AVPR V2 tail fused to the TEV protease (TEVp). A BFP is fused to the C-terminus of GPCR-V2-TEVp via a p2A sequence for visualization. These plasmids are co-transfected into a stable reporter cell line containing GFP under control of the Doxycycline (Dox)-inducible TRE3G promoter. The size of the GPCR fusion protein is 2.7kb. (b) The plasmids for the GPCR-CRISPR ChaCha system. The size of the GPCR fusion protein is 8.4kb.

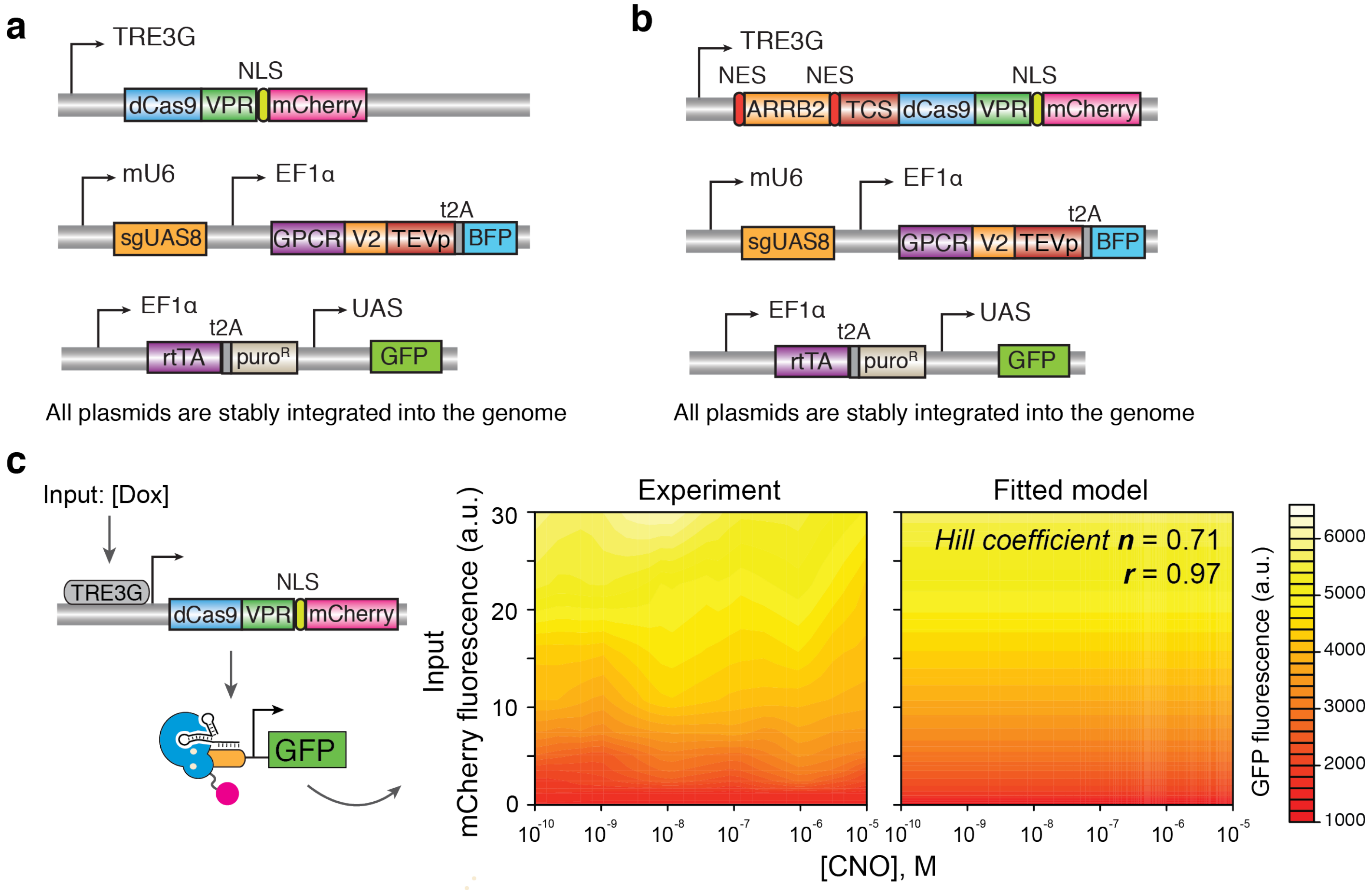

Supplementary Figure 2 | Modeling to estimate the number of dCas9 molecules released per receptor. (**a**) The plasmid system for gene activation using free dCas9-VPR. (**b**) The plasmid system for gene activation using the ChaCha system. (**c**) Measurement and modeling EGFP reporter activation via Dox-inducible dCas9-VPR-mCherry. Left, schematic of the HEK293T cell line containing genomically integrated lentiviral pTRE3G-dCas9-VPR-mCherry, pUAS-GFP-pEF1α-rtTA-p2A-puroR, and pU6-sgUAS8, see **Supplementary Table 1**. Dox induces expression of dCas9-VPR-mCherry, which with sgUAS8 binds and activates the GFP reporter. Plots demonstrate GFP fluorescence (a.u.) as a function of mCherry fluorescence (a.u., y-axis; a surrogate for Dox), and CNO concentration (M, x-axis). Both the experimental (middle) and fitted-model plots (right) demonstrate a Dox-dependent, CNO-independent graded GFP response. Model fitting using nonlinear regression indicates $n < 1$ with correlation coefficient r = 0.97 (right). See **Methods** for the detailed model.

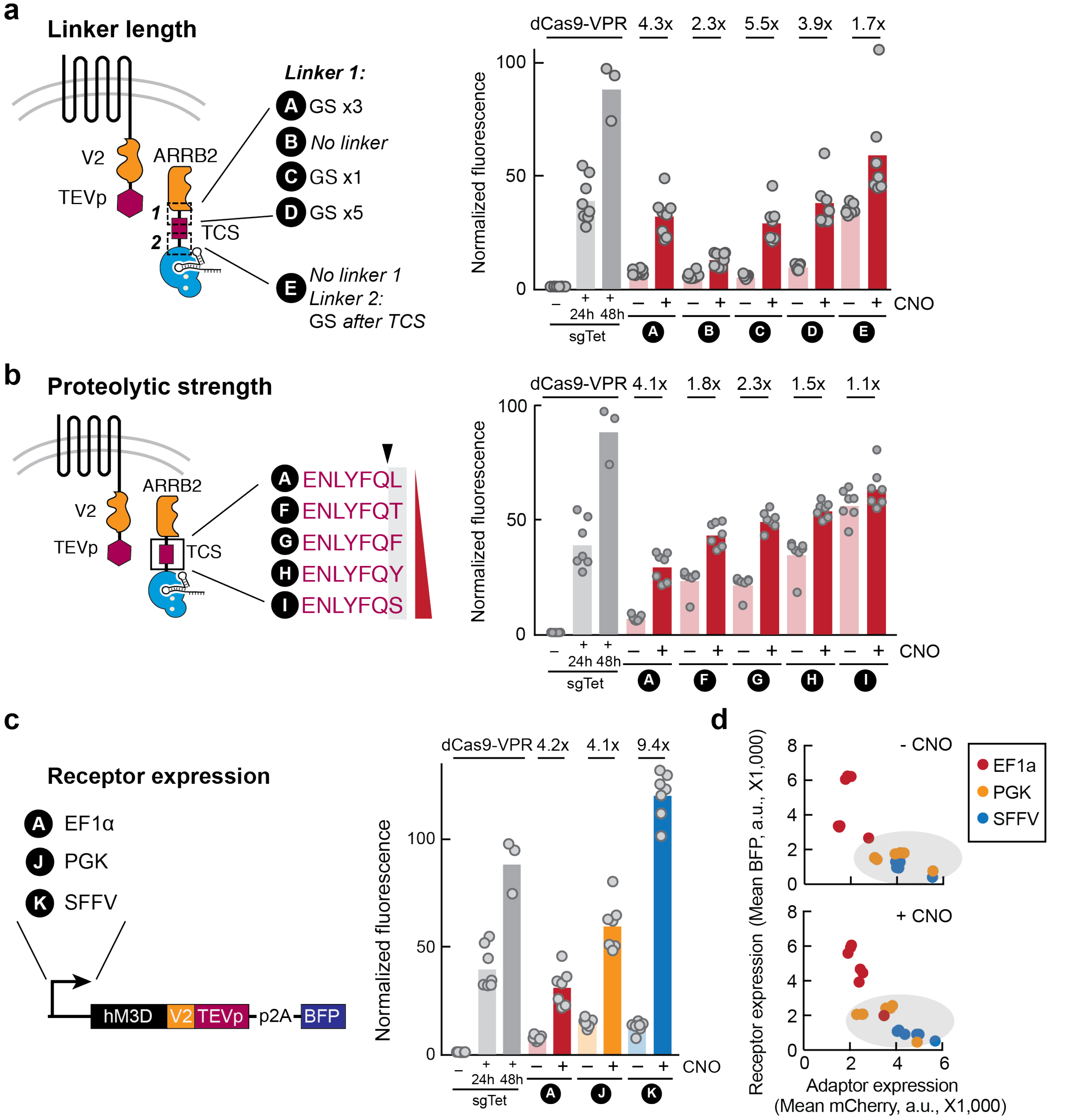

Supplementary Figure 3 | Characterizing design parameters of hM3D-CRISPR ChaCha. (**a**) Left, flexible glycine-serine (GS) linkers (variants **A – E**) before or after TEV cleavage site (TCS) were varied as indicated. Right, comparison of GFP activation for variants **A – E**, after 1 day of +/- CNO treatment. (**b**) Left, TCS variants **F – I** with increasing proteolytic strengths. Red wedge indicates increasing proteolytic cleavage efficiency. Right, comparison of GFP activation for these variants after 1 day of +/- CNO treatment. (**c**) Comparison of

GFP activation by receptor promoter variants (**A, J, K**) as indicated, after 1 day of +/- CNO treatment. The grey bars represent GFP activation using free dCas9-VPR with or without a targeting sgRNA (sgTet) for 24h or 48h. +/- indicates with or without CNO. The fold of activation displayed on top compares +/-CNO conditions. Data was normalized to the free dCas9-VPR without a targeting sgRNA. **(d)** Quantification of receptor (GPCR-V2-TEVp) and adaptor (ARRB2-TCS-dCas9-VPR) expression levels for BFP (receptor), mCherry (adaptor), and GFP (reporter) triple positive cells from experiments in (**c**) for with and without CNO. The data represent three independent experiments with technical replicates. The grey shaded region shows samples with a higher ratio of adaptor to receptor expression, which also show a higher dynamic range for gene activation in (**c**).

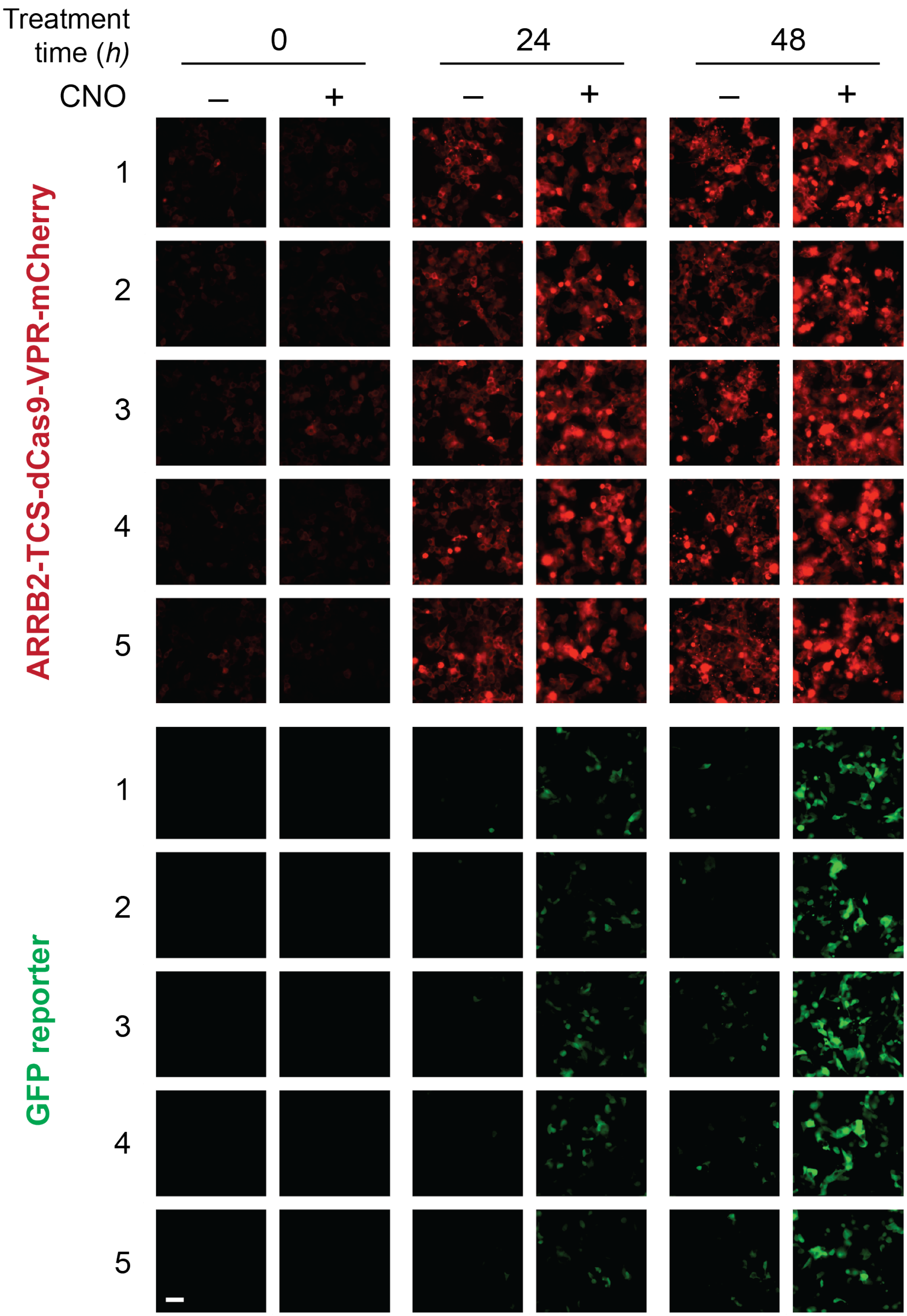

Supplementary Figure 4 | Additional fields of view from time-lapse microscopy of hM3D-CRISPR ChaCha activation in live HEK293T cells. Related to **Fig. 2a** and **Supplementary Movies 1-3**.

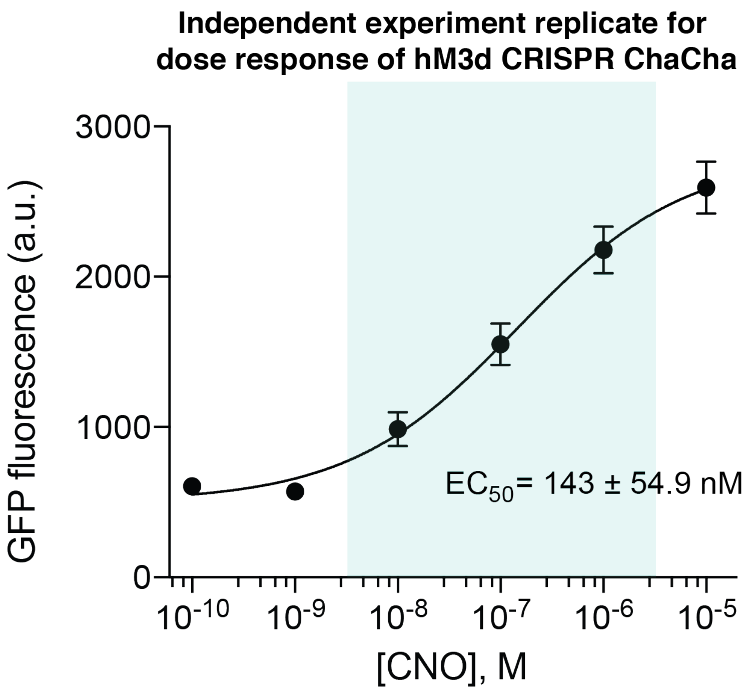

Supplementary Figure 5 | Independent experimental replicate of the measured CNO dose response curve for the hM3D-CRISPR ChaCha. Related to **Fig. 2b**.

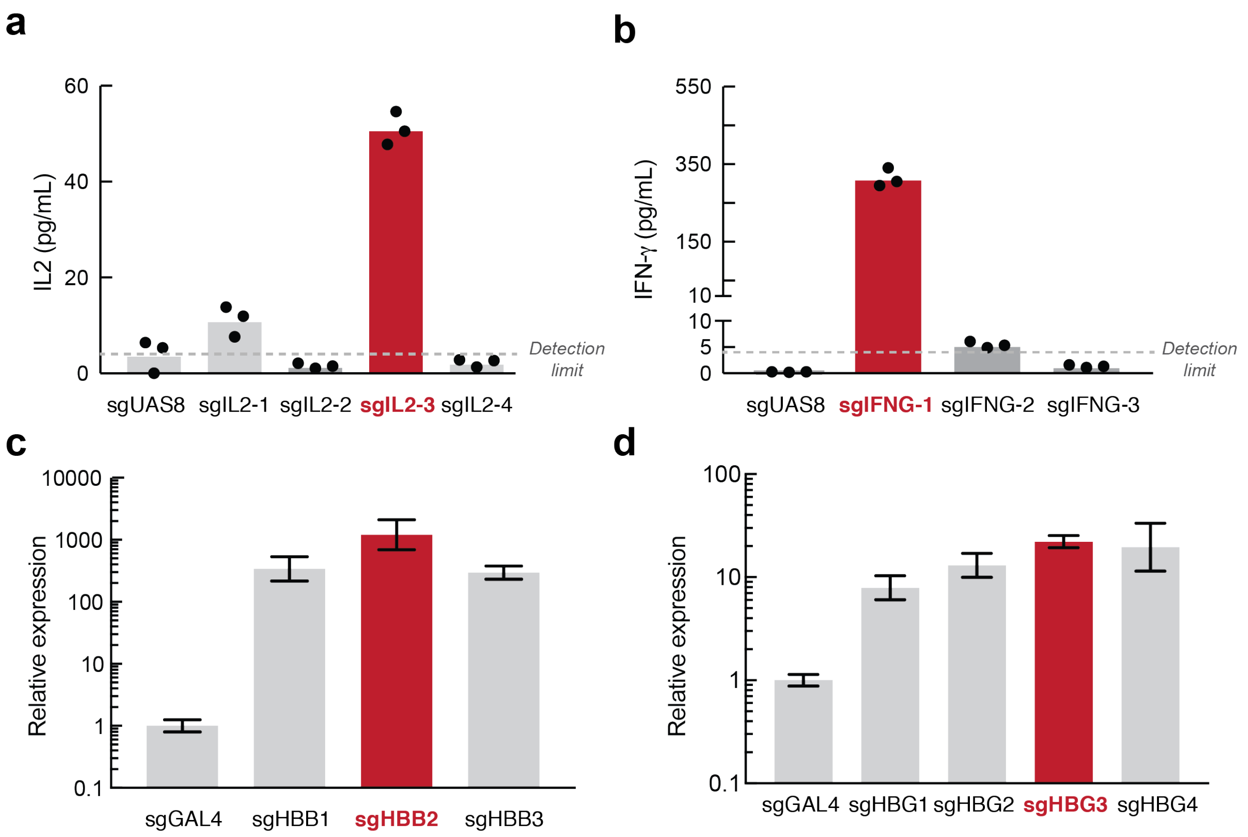

Supplementary Figure 6 | Characterization of sgRNA efficiency for target gene activation. (a-b) Screen of sgRNAs for IL2 and IFN-γ cytokine activation and secretion using ELISA. sgUAS8 is used as a non-targeting control. The best performing sgRNAs (red) are used for experiments in the main text. The dotted line represents the detection limit of ELISA (4 pg/mL). **(c-d)** Screen of sgRNAs for HBB and HBG activation using qPCR. sgGAL4 was used as a non-targeting control. The best performing sgRNAs (red) were used in the main text.

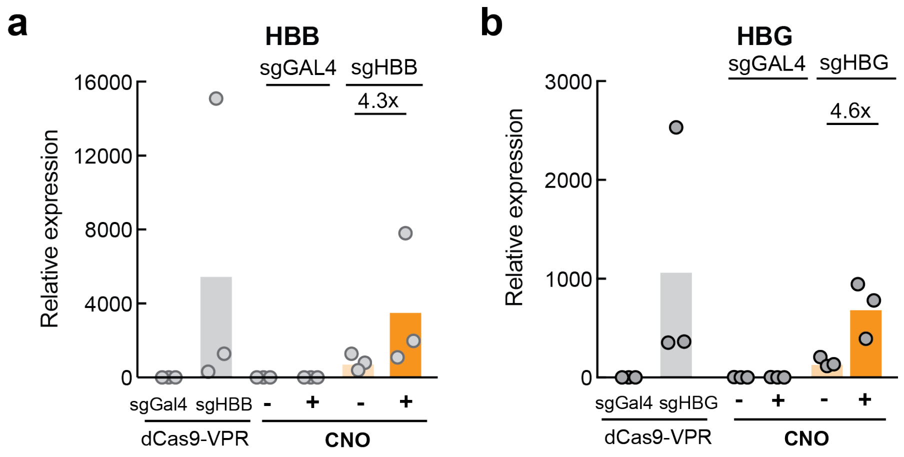

Supplementary Figure 7 | (**a-b**) Activation of HBB and HBG using the hM3D-CRISPR ChaCha system measured by quantitative PCR (qPCR) with or without 10 μM CNO compared to positive and negative controls using free dCas9-VPR. Related to **Fig. 3b**.

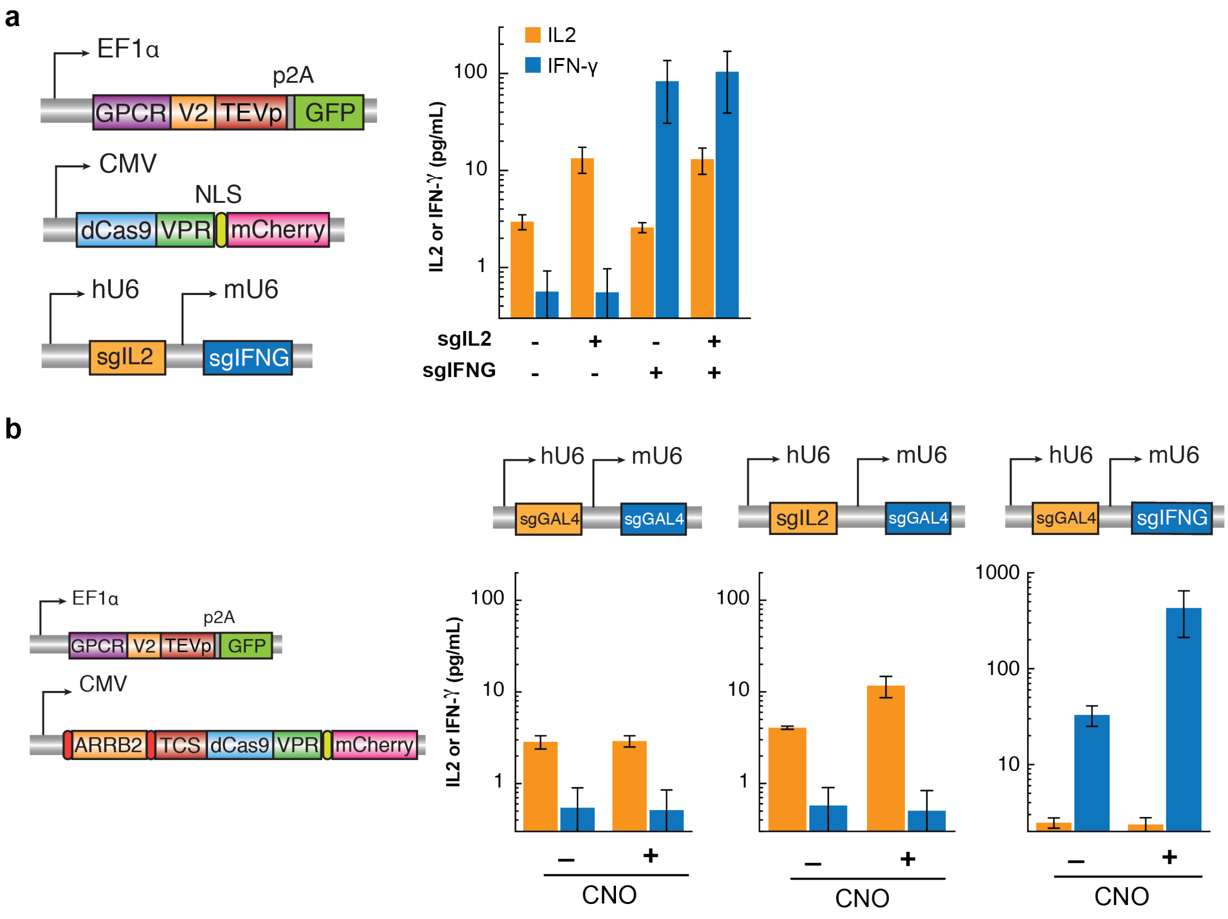

Supplementary Figure 8 | Control experiments for the multiplexed activation of two genes using CRISPR ChaCha. (**a**) The control experiment for the multiplexed activation of two endogenous genes using free dCas9-VPR. The plasmids used for the experiments are shown on the top. Measurement of IL2 (orange) and IFN-γ (blue) by ELISA with different combinations of targeting sgRNAs is shown on the bottom. (**b**) The control experiment for the multiplexed activation of two endogenous genes using a non-targeting sgRNA (sgGAL4) or a single sgRNA for IL2 (sgIL2) or IFN-γ (sgINFG). The plasmids used for the experiments are shown on the top. Measurement of IL2 and IFN-γ expression and secretion by ELISA with and without CNO is shown in the bottom diagrams. Related to **Fig. 3c.**

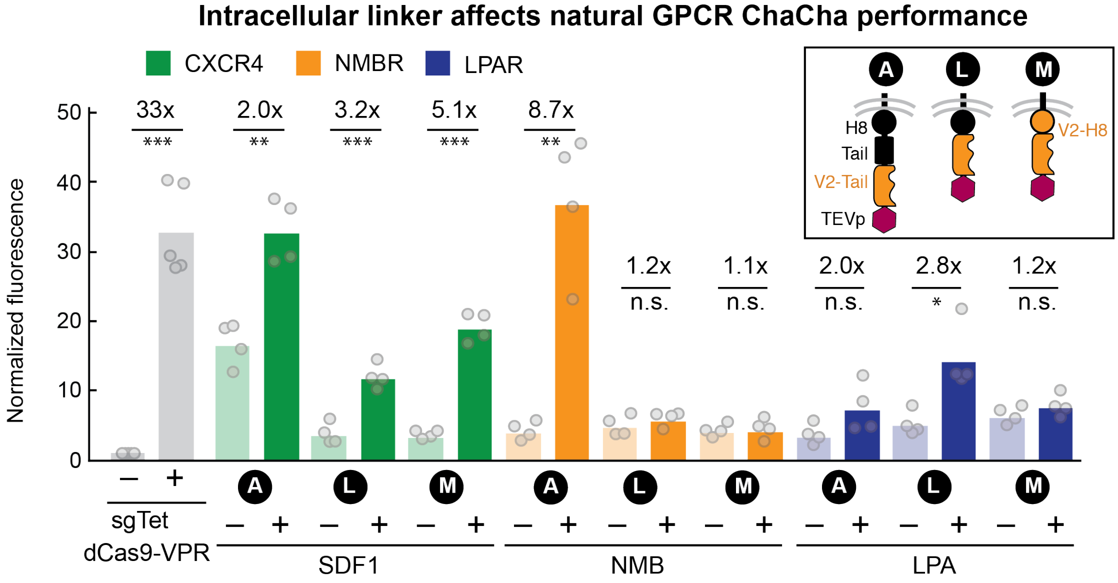

Supplementary Figure 9 | The intracellular linker affects natural GPCR ChaCha performance. Inset: variant A is the original ChaCha architecture; variant L is a C-terminal tail replacement with that of V2, and variant M is a C-terminal tail and H8 domain replacement to V2. The data shows GFP reporter activation measured by flow cytometry for each variant with (+) or without (-) of cognate ligands for 1 day. The free dCas9-VPR with or without a targeting sgRNA (sgTet) was used as controls. Dots represent biological measurements, and bars represent means from 2 independent experiments. The fold of activation displayed on top of bars compares +/- treatment for GPCR variants, or with or without a targeting sgRNA for the free dCas9-VPR.

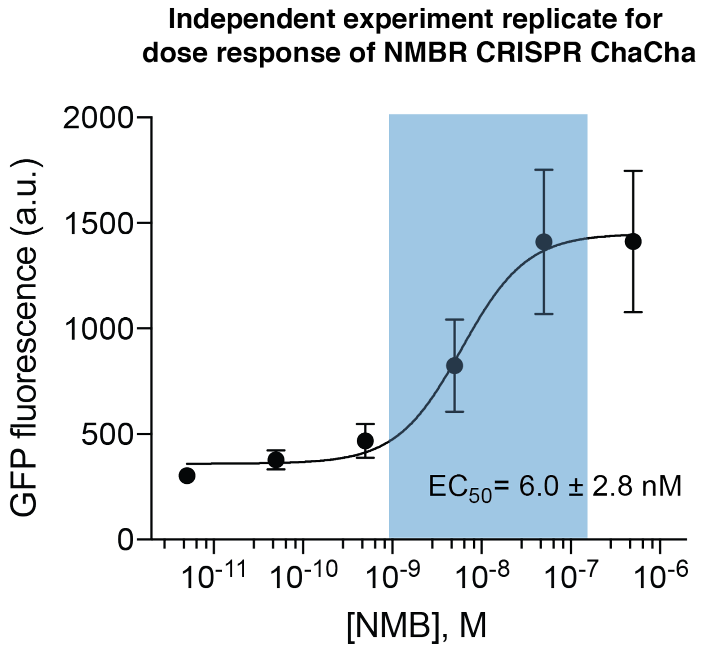

Supplementary Figure 10 | Independent experimental replicate of the measured neuromedin B dose response curve for NMBR-CRISPR ChaCha. Related to **Fig. 4c**.

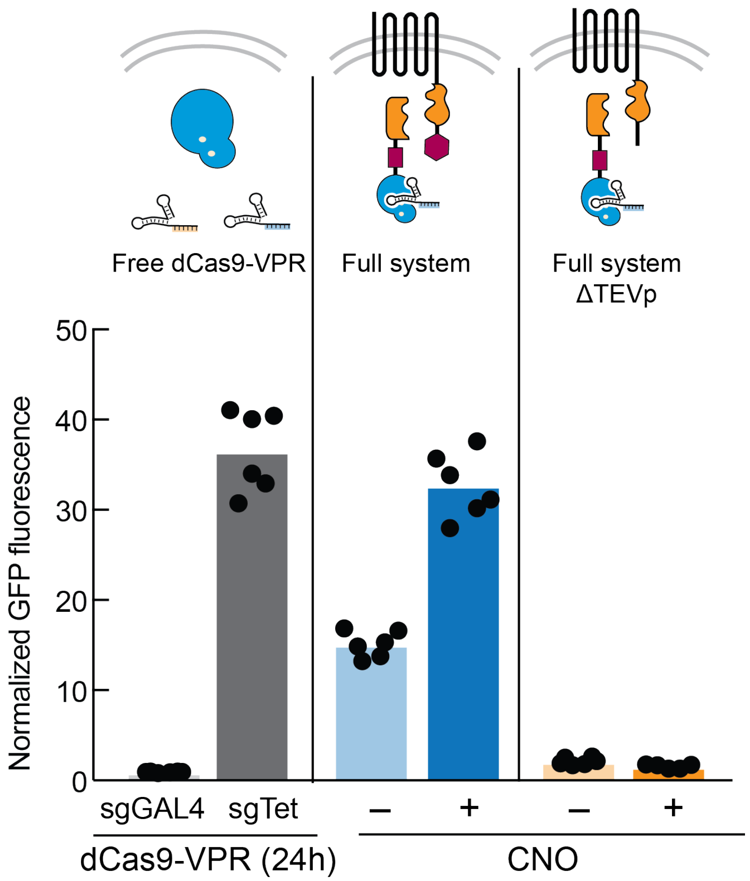

Supplementary Figure 11 | Experimental data on characterizing the leaky activation of the ChaCha System. (**a**) Expression of GFP for free dCas9-VPR with a non-targeting sgRNA (sgGAL4) or a targeting sgRNA (sgTet) 24 h after transfection (grey, left), the full ChaCha system with or without CNO (blue, middle), and the full ChaCha system with the TEVp removed from the C-terminus of the receptor (orange, right). The system used in each group is shown on top of the bars. The GFP fluorescence is normalized to the free dCas9-VPR with a non-targeting sgGAL4. Bars represent the mean, and data shown is from two independent experiments with three technical replicates.

Supplementary Table 1. Plasmid constructs used in this study listed by figure.

ChaCha variants are indicated in parentheses. Constructs used to generate the two reporter cell lines are described in the **Methods**.

Figure	Constructs	Plasmid ID
**1b**	pcDNA3: dCas9-VPR-mCherry	pSLQ3958
	pHR: U6-sgTET EF1a-hM3D-V2-TEV-p2a-BFP (A)	pSLQ3956
	pHR: EF1a-hM3D-V2-TEV-p2a-BFP (A)	pSLQ5768
	pcDNA3: hM3D-V2-TCS-dCas9-NLS-VPR-mCherry	pSLQ3875
	pHR: U6-sgTET EF1a-NES-ARRB2-NES-TEV-p2a-BFP	pSLQ5781
	pcDNA3: NES-ARRB2-NES-TCS-dCas9-VPR-mCherry (A)	pSLQ3960
**1c**	pHR: U6-sgUAS8 EF1a-hM3D-V2-TEV-p2a-BFP (A)	pSLQ5779
	pHR: TRE3G-NES-ARRB2-NES-TCS-dCas9-VPR-mCherry (A)	pSLQ5751
	pHR: EF1a -rttA-p2A-Puro – WPRE UAS -GFP – bGH-pA.	pSLQ5738
**2a**	pHR: U6-sgTET SFFV-hM3D-V2-TEV-p2a-BFP (K)	pSLQ5777
	pcDNA3: NES-ARRB2-NES-TCS-dCas9-VPR-mCherry (A)	pSLQ3960
**2b**	pHR: U6-sgTET SFFV-hM3D-V2-TEV-p2a-BFP (K)	pSLQ5777
	pcDNA3: NES-ARRB2-NES-TCS-dCas9-VPR-mCherry (A)	pSLQ3960
**2c**	pHR: U6-sgUAS8 EF1a-hM3D-V2-TEV-p2a-BFP (A)	pSLQ5779
	pHR: TRE3G-NES-ARRB2-NES-TCS-dCas9-VPR-mCherry (A)	pSLQ5751
	pHR: EF1a -rttA-p2A-Puro – WPRE UAS -GFP – bGH-pA.	pSLQ5738
**3a**	pcDNA3: dCas9-VPR-mCherry	pSLQ3958
	pHR: EF1a-hM3D-V2-linker-TEV-p2a-EGFP (A)	pSLQ5767
	pcDNA3: NES-ARRB2-NES-TCS-dCas9-VPR-mCherry (A)	pSLQ3960
	pHR: U6-sgGal4 EF1a-puro-t2a-BFP	-
	pHR: U6-sgIL2 EF1a-puro-t2a-BFP	-
	pHR: U6-sgIFNG EF1a-puro-t2a-BFP	-
**3b**	pcDNA3: dCas9-VPR-mCherry	pSLQ3958
	pHR: EF1a-hM3D-V2-linker-TEV-p2a-EGFP (A)	pSLQ5767
	pcDNA3: NES-ARRB2-NES-TCS-dCas9-VPR-mCherry (A)	pSLQ3960
	pHR: U6-sgGal4 EF1a-puro-t2a-BFP	-
	pHR: U6-sgHBB EF1a-puro-t2a-BFP	-
	pHR: U6-sgHBG EF1a-puro-t2a-BFP	-
**3c**	pcDNA3: dCas9-VPR-mCherry	pSLQ3958
	pHR: EF1a-hM3D-V2-linker-TEV-p2a-EGFP (A)	pSLQ5767
	pcDNA3: NES-ARRB2-NES-TCS-dCas9-VPR-mCherry (A)	pSLQ3960
	pHR: hU6-sgGal4 mU6-sgGal4 EF1a-puro-t2a-BFP	-
	pHR: hU6-sgIL2 mU6-sgIFNG EF1a-puro-t2a-BFP	-
**4b.**	pHR: U6-sgTET EF1a-KORD-V2-TEV-p2a-BFP (A)	pSLQ5736
	pHR: U6-sgTET EF1a-LPAR1-V2-TEV-p2a-BFP (L)	pSLQ5774
	pHR: U6-sgTET EF1a-CXCR4-V2-TEV-p2a-BFP (M)	pSLQ5771

	pHR: U6-sgTET EF1a-NMBR-V2-TEV-p2a-BFP (A)	pSLQ3780
	pHR: U6-sgTET EF1a-ADRB2-V2-TEV-p2a-BFP (A)	pSLQ5901
	pHR: U6-sgTET EF1a-AVPR2-V2-TEV-p2a-BFP (A)	pSLQ5908
	pHR: U6-sgTET EF1a-TRHR-V2-TEV-p2a-BFP (A)	pSLQ5909
	pcDNA3: NES-ARRB2-NES-TCS-dCas9-VPR-mCherry (A)	pSLQ3960
**4c**	pcDNA3: NES-ARRB2-NES-TCS-dCas9-VPR-mCherry (A)	pSLQ3960
	pHR: U6-sgTET EF1a-NMBR-V2-TEV-p2a-BFP (A)	pSLQ3780
**4d**	pcDNA3: dCas9-VPR-mCherry	pSLQ3958
	pHR: EF1a-NMBR-V2-linker-TEV-p2a-EGFP (A)	pSLQ5815
	pcDNA3: NES-ARRB2-NES-TCS-dCas9-VPR-mCherry (A)	pSLQ3960
	pHR: U6-sgGal4 EF1a-puro-t2a-BFP	-
	pHR: U6-sgIFNG EF1a-puro-t2a-BFP	-
	**Supplementary Figures**	
**S2c**	pHR: U6-sgUAS8 EF1a-hM3D-V2-TEV-p2a-BFP (A)	pSLQ5779
	pHR: TRE3G-dCas9-VPR-mCherry	pSLQ5750
	pHR: EF1a -rttA-p2A-Puro – WPRE UAS -GFP – bGH-pA.	pSLQ5738
**S3a**	pcDNA3: dCas9-VPR-mCherry	pSLQ3958
	pHR: U6-sgTET EF1a-hM3D-V2-TEV-p2a-BFP (A)	pSLQ3956
	pHR: EF1a-hM3D-V2-TEV-p2a-BFP (A)	pSLQ5768
	pcDNA3: NES-ARRB2-NES-TCS-dCas9-VPR-mCherry (A)	pSLQ3960
	pcDNA3: NES-ARRB2-NES-TCS-dCas9-VPR-mCherry (B)	pSLQ5740
	pcDNA3: NES-ARRB2-NES-TCS-dCas9-VPR-mCherry (C)	pSLQ5741
	pcDNA3: NES-ARRB2-NES-TCS-dCas9-VPR-mCherry (D)	pSLQ5744
	pcDNA3: NES-ARRB2-NES-TCS-dCas9-VPR-mCherry (E)	pSLQ5745
**S3b**	pcDNA3: dCas9-VPR-mCherry	pSLQ3958
	pHR: U6-sgTET EF1a-hM3D-V2-TEV-p2a-BFP (A)	pSLQ3956
	pHR: EF1a-hM3D-V2-TEV-p2a-BFP (A)	pSLQ5768
	pcDNA3: NES-ARRB2-NES-TCS-dCas9-VPR-mCherry (A)	pSLQ3960
	pcDNA3: NES-ARRB2-NES-TCS-dCas9-VPR-mCherry (F)	pSLQ5727
	pcDNA3: NES-ARRB2-NES-TCS-dCas9-VPR-mCherry (G)	pSLQ5728
	pcDNA3: NES-ARRB2-NES-TCS-dCas9-VPR-mCherry (H)	pSLQ5729
	pcDNA3: NES-ARRB2-NES-TCS-dCas9-VPR-mCherry (I)	pSLQ5730
**S3c**	pcDNA3: dCas9-VPR-mCherry	pSLQ3958
	pHR: U6-sgTET EF1a-hM3D-V2-TEV-p2a-BFP (A)	pSLQ3956
	pHR: EF1a-hM3D-V2-TEV-p2a-BFP (A)	pSLQ5768
	pcDNA3: NES-ARRB2-NES-TCS-dCas9-VPR-mCherry (A)	pSLQ3960
	pHR: U6-sgTET PGK-hM3D-V2-TEV-p2a-BFP (J)	pSLQ5776
	pHR: U6-sgTET SFFV-hM3D-V2-TEV-p2a-BFP (K)	pSLQ5777
**S4**	pHR: U6-sgTET SFFV-hM3D-V2-TEV-p2a-BFP (K)	pSLQ5777
	pcDNA3: NES-ARRB2-NES-TCS-dCas9-VPR-mCherry (A)	pSLQ3960
**S5**	pHR: U6-sgTET SFFV-hM3D-V2-TEV-p2a-BFP (K)	pSLQ5777
	pcDNA3: NES-ARRB2-NES-TCS-dCas9-VPR-mCherry (A)	pSLQ3960

**S6a**	pcDNA3: dCas9-VPR-mCherry	pSLQ3958
	pHR: U6-sgUAS8 EF1a-puro-t2a-BFP	-
	pHR: U6-sgIL2-1 EF1a-puro-t2a-BFP	-
	pHR: U6-sgIL2-2 EF1a-puro-t2a-BFP	-
	pHR: U6-sgIL2-3 EF1a-puro-t2a-BFP	-
	pHR: U6-sgIL2-4 EF1a-puro-t2a-BFP	-
**S6b**	pcDNA3: dCas9-VPR-mCherry	pSLQ3958
	pHR: U6-sgIFNG-1 EF1a-puro-t2a-BFP	-
	pHR: U6-sgIFNG-2 EF1a-puro-t2a-BFP	-
	pHR: U6-sgIFNG-3 EF1a-puro-t2a-BFP	-
**S7**	pcDNA3: dCas9-VPR-mCherry	pSLQ3958
	pHR: U6-sgGAL4 EF1a-puro-t2a-BFP	-
	pHR: U6-sgHBB2 EF1a-puro-t2a-BFP	
	pHR: U6-sgHBG3 EF1a-puro-t2a-BFP	
	pHR: EF1a-hM3D-V2-linker-TEV-p2a-EGFP (A)	pSLQ5767
	pcDNA3: NES-ARRB2-NES-TCS-dCas9-VPR-mCherry (A)	pSLQ3960
**S8a**	pcDNA3: dCas9-VPR-mCherry	pSLQ3958
	pHR: hU6-sgGal4 mU6-sgGal4 EF1a-puro-t2a-BFP	
	pHR: hU6-sgIL2 mU6-sgGal4 EF1a-puro-t2a-BFP	
	pHR: hU6-sgGal4 mU6-sgIFNG EF1a-puro-t2a-BFP	
	pHR: hU6-sgIL2 mU6-sgIFNG EF1a-puro-t2a-BFP	
	pHR: EF1a-hM3D-V2-linker-TEV-p2a-EGFP (A)	pSLQ5767
**S8b**	pcDNA3: NES-ARRB2-NES-TCS-dCas9-VPR-mCherry (A)	pSLQ3960
	pHR: hU6-sgGal4 mU6-sgGal4 EF1a-puro-t2a-BFP	
	pHR: hU6-sgIL2 mU6-sgGal4 EF1a-puro-t2a-BFP	
	pHR: hU6-sgGal4 mU6-sgIFNG EF1a-puro-t2a-BFP	
	pHR: hU6-sgIL2 mU6-sgIFNG EF1a-puro-t2a-BFP	
**S9**	pcDNA3: dCas9-VPR-mCherry	pSLQ3958
	pHR: EF1a-hM3D-V2-TEV-p2a-BFP (A)	pSLQ5768
	pcDNA3: NES-ARRB2-NES-TCS-dCas9-VPR-mCherry (A)	pSLQ3960
	pHR: U6-sgTET EF1a-CXCR4-V2-TEV-p2a-BFP (A)	pSLQ3779
	pHR: U6-sgTET EF1a-CXCR4-V2-TEV-p2a-BFP (L)	pSLQ5770
	pHR: U6-sgTET EF1a-CXCR4-V2-TEV-p2a-BFP (M)	pSLQ5771
	pHR: U6-sgTET EF1a-NMBR-V2-TEV-p2a-BFP (A)	pSLQ3780
	pHR: U6-sgTET EF1a-NMBR-V2-TEV-p2a-BFP (L)	pSLQ5772
	pHR: U6-sgTET EF1a-NMBR-V2-TEV-p2a-BFP (M)	pSLQ5773
	pHR: U6-sgTET EF1a-LPAR1-V2-TEV-p2a-BFP (A)	pSLQ3781
	pHR: U6-sgTET EF1a-LPAR1-V2-TEV-p2a-BFP (L)	pSLQ5774
	pHR: U6-sgTET EF1a-LPAR1-V2-TEV-p2a-BFP (M)	pSLQ5775
**S10**	pcDNA3: NES-ARRB2-NES-TCS-dCas9-VPR-mCherry (A)	pSLQ3960
	pHR: U6-sgTET EF1a-NMBR-V2-TEV-p2a-BFP (A)	pSLQ3780
**S11**	pcDNA3: dCas9-VPR-mCherry	pSLQ3958
	pHR: U6-sgTET EF1a-puro-t2a-BFP	

	pHR: U6-sgGal4 EF1a-puro-t2a-BFP	
	pHR: U6-sgTET EF1a-hM3D-V2-TEV-p2a-BFP (A)	pSLQ3956
	pcDNA3: NES-ARRB2-NES-TCS-dCas9-VPR-mCherry (A)	pSLQ3960
	pHR: U6-sgTET EF1a-hM3D-V2-p2a-BFP (A)	pSLQ5725

Supplementary Table 2: Sequences for *S. pyogenes* sgRNAs used in the study.

Name	Spacer Sequence
sgTET	GTACGTTCTCTATCACTGATA
sgGAL4	GAGCACTGTCCTCCGAACGT
sgUAS8	GTACTCCGACCTCTAGTGT
sgIL2-1	GTGATTAAAGAGAGTGATA
sgIL2-2	GCAATTTATACTGTTAATTC
sgIL2-3	GTTACATTAGCCCACACTT
sgIL2-4	GAAAAACTGTTTCATACAGA
sgIFNG-1	GAGATGGTGACAGATAGGCA
sgIFNG-2	GAATGGCACAGGTGGGCATAA
sgIFNG-3	GTACCTCCCCACTTCGCCC
sgHBB1	GTGGAGCCACACCCTAGGGT
sgHBB2	GAAGAGCCAAGGACAGGTA
sgHBB3	GTACCTGTCCTTGGCTCTTC
HBG1	GTCAAGGCAAGGCTGGCCAACCCA
HBG2	GCTATTGGTCAAGGCAAGGC
HBG3	GCTAAACTCCACCCATGGGT
HBG4	GATATTTGCATTGAGATAGTG

Supplementary Table 3. cDNA resources used for receptor cloning.

**Receptor cDNA**	**Repository**	**Catalogue No.**
hM3D	Addgene	45547
KORD	Addgene	65417
CXCR4	Addgene	66262
LPAR1	Addgene	66418
NMBR	Addgene	66445
ADRB2	Addgene	66220
TRHR	cDNA Resource Center (www.cdna.org)	TRHR000000
AVPR2	Addgene	66227

Supplementary Table 4. GPCR ligands used.

Receptor	Ligand	Concentration	Manufacturer	Catalogue No.	Media Conditions
hM3D	Clozapine N-Oxide (CNO)	20 μM	Sigma	C0832	Full Serum
KORD	Salvinorin B (SALB)	2 μM	Tocris	5611	Full Serum
CXCR4	Stromal-cell Derived Factor 1 (SDF1)	0.05 μM	BioLegend	581204	Serum Free
LPAR1	Lysophosphatidic acid (LPA)	10 μM	Santa Cruz Biotech	sc-201053	Serum Free
NMBR	Neuromedin B (NMB)	1 μM	Tocris	1908	Serum Free
ADRB2	Isoproterenol HCL (ISO)	10 μM	Tocris	1747	Full Serum
TRHR	Thyrotropin Releasing Horomone (TRH)	10 μM	Sigma	P1319-50MG	Full Serum
AVPR2	Arg8- Vassopressin (AVP)	10 μM	Tocris	2935	Full Serum

Supplementary Table 5. Primers used for qPCR

qPCR Primer	Sequence
GAPDH.F	TGCCAAATATGATGACATCAAGAA
GAPDH.R	GGAGTGGGTGTCGCTGTTGC
HBG.F	GCTGAGTGAACTGCACTGTGA
HBG.R	GAATTCTTTGCCGAAATGGA
HBB.F	GCACGTGGATCCTGAGAACT
HBB.R	ATTGGACAGCAAGAAAGCGAG

Supplementary Table 6. Statistical Analyses of Experimental Data

See Figure Legends and **Methods** for descriptions of statistical tests used. Fold changes are mean values that are reflected in the figures. Statistically significant differences (P < 0.05) are **bolded.**

Figure	Condition 1	Condition 2	Fold	P-value
1b	dCas9-VPR (N)	dCas9-VPR (T)	31	**0.002**
	hM3D Tango (- CNO)	hM3D Tango (+ CNO)	1.2	0.216
	hM3D ChaCha (- CNO)	hM3D ChaCha (+ CNO)	4.4	**<0.001**
3a	dCas9-VPR (sgGal4)	dCas9-VPR (sgIL2)	>11*	**<0.001**
	hM3D ChaCha (sgGal4, - CNO)	hM3D ChaCha (sgGal4, + CNO)	b.d.l.*	-
	hM3D ChaCha (sgIL2, - CNO)	hM3D ChaCha (sgIL2, + CNO)	3.4	**<0.001**
	dCas9-VPR (sgGal4)	dCas9-VPR (sgIFNG)	>92*	**<0.001**
	hM3D ChaCha (sgGal4, - CNO)	hM3D ChaCha (sgGal4, + CNO)	b.d.l.*	-
	hM3D ChaCha (sgIFNG, - CNO)	hM3D ChaCha (sgIFNG, + CNO)	11	**<0.001**
3b	hM3D ChaCha (sgGal4, - CNO)	hM3D ChaCha (sgGal4, + CNO)	1.59	-
	hM3D ChaCha (sgHBB, - CNO)	hM3D ChaCha (sgHBB, + CNO)	4.3	0.351
	hM3D ChaCha (sgGal4, - CNO)	hM3D ChaCha (sgGal4, + CNO)	1.25	-
	hM3D ChaCha (sgHBG, - CNO)	hM3D ChaCha (sgHBG, + CNO)	4.6	0.0743
3c	hM3D ChaCha (sgIL2/sgIFNG, - CNO)	hM3D ChaCha (sgIL2/sgIFNG,, + CNO)	3.3	**<0.0001**
	hM3D ChaCha (sgIL2/sgIFNG, - CNO)	hM3D ChaCha (sgIL2/sgIFNG,, + CNO)	11	**0.0002**
4a	KORD ChaCha (-SALB)	KORD ChaCha (+SALB)	6.4	**<0.001**
	LPAR1 ChaCha (- LPA) Variant L	hM3D ChaCha (+ LPA) Variant L	2.8	**0.024**
	CXCR4 ChaCha (- SDF1) Variant M	CXCR4 ChaCha (+ SDF1) Variant M	5.1	**<0.001**
	NMBR ChaCha (- NMB)	NMBR ChaCha (+ NMB)	8.7	**0.007**

	Variant A	Variant A		
	ADRB2 ChaCha (-ISO)	ADRB2 ChaCha (+ISO)	4.0	**0.0027**
	AVPR2 ChaCha (-AVP)	AVPR2 ChaCha (+AVP)	3.1	**<0.0001**
	TRHR ChaCha (-TRH)	TRHR ChaCha (+TRH)	3.9	**<0.0001**
	dCas9-VPR (sgGal4)	dCas9-VPR (sgIFNG)	>97	**<0.001**
4d	NMBR ChaCha (sgGal4, - NMB)	NMBR ChaCha (sgGal4, + NMB)	n.d.*	-
	NMBR ChaCha (sgIFNG, - NMB)	NMBR ChaCha (sgIFNG, + NMB)	20	**0.009**
S1a	dCas9-VPR (N)	dCas9-VPR (T), 24 hrs	39	**<0.0001**
	dCas9-VPR (N)	dCas9-VPR (T), 48 hrs	89	**0.0067**
	hM3D ChaCha Variant A (-CNO)	hM3D ChaCha Variant A (+CNO)	4.3	**<0.0001**
	hM3D ChaCha Variant B (-CNO)	hM3D ChaCha Variant B (+CNO)	2.3	**0.0001**
	hM3D ChaCha Variant C (-CNO)	hM3D ChaCha Variant C (+CNO)	5.5	**0.0003**
	hM3D ChaCha Variant D (-CNO)	hM3D ChaCha Variant D (+CNO)	3.9	**0.0004**
	hM3D ChaCha Variant E (-CNO)	hM3D ChaCha Variant E (+CNO)	1.7	**0.0278**
S1b	dCas9-VPR (N)	dCas9-VPR (T), 24 hrs	39	**<0.0001**
	dCas9-VPR (N)	dCas9-VPR (T), 48 hrs	89	**0.0067**
	hM3D ChaCha Variant A (-CNO)	hM3D ChaCha Variant A (+CNO)	4.1	**<0.0001**
	hM3D ChaCha Variant F (-CNO)	hM3D ChaCha Variant F (+CNO)	1.8	**<0.0001**
	hM3D ChaCha Variant G (-CNO)	hM3D ChaCha Variant G (+CNO)	2.3	**0.0002**

	hM3D ChaCha Variant H (-CNO)	hM3D ChaCha Variant H (+CNO)	1.5	**<0.0001**
	hM3D ChaCha Variant I (-CNO)	hM3D ChaCha Variant I (+CNO)	1.1	0.1124
S1c	dCas9-VPR (N)	dCas9-VPR (T), 24 hrs	39	**<0.0001**
	dCas9-VPR (N)	dCas9-VPR (T), 48 hrs	89	**0.0067**
	hM3D ChaCha Variant A (-CNO)	hM3D ChaCha Variant A (+CNO)	4.2	**0.0003**
	hM3D ChaCha Variant J (-CNO)	hM3D ChaCha Variant J (+CNO)	4.1	**<0.0001**
	hM3D ChaCha Variant K (-CNO)	hM3D ChaCha Variant K (+CNO)	9.4	**<0.0001**
S6a	dCas9-VPR (sgUAS8)	dCas9-VPR (sgIL2-1)	2.1	-
	dCas9-VPR (sgUAS8)	dCas9-VPR (sgIL2-2)	b.d.l.*	-
	dCas9-VPR (sgUAS8)	dCas9-VPR (sgIL2-3)	9.7	-
	dCas9-VPR (sgUAS8)	dCas9-VPR (sgIL2-4)	b.d.l.*	-
S6b	dCas9-VPR (sgUAS8)	dCas9-VPR (sgIFNG-1)	78	-
	dCas9-VPR (sgUAS8)	dCas9-VPR (sgIFNG-2)	-	-
	dCas9-VPR (sgUAS8)	dCas9-VPR (sgIFNG-3)	b.d.l.*	-
S6c	dCas9-VPR (sgGAL4)	dCas9-VPR (sgHBB-1)	340	-
	dCas9-VPR (sgGAL4)	dCas9-VPR (sgHBB-2)	1204	-
	dCas9-VPR (sgGAL4)	dCas9-VPR (sgHBB-3)	292	-
	dCas9-VPR (sgGAL4)	dCas9-VPR (sgHBG-1)	7.8	-
	dCas9-VPR (sgGAL4)	dCas9-VPR (sgHBG-2)	12.9	-
	dCas9-VPR (sgGAL4)	dCas9-VPR (sgHBG-3)	22.1	-
	dCas9-VPR (sgGAL4)	dCas9-VPR (sgHBG-4)	19.5	-
S7a	dCas9 VPR (sgGal4)	dCas9 VPR (sgHBB)	5560	0.0783
	hM3D ChaCha (sgGal4, - CNO)	hM3D ChaCha (sgGal4, + CNO)	-	-
	hM3D ChaCha	hM3D ChaCha	4.3	0.0510

	(sgHBB, - CNO)	(sgHBB, + CNO)		
S7b	dCas9 VPR (sgGal4)	dCas9 VPR (sgHBG)	1083	**0.0323**
	hM3D ChaCha (sgGal4, - CNO)	hM3D ChaCha (sgGal4, + CNO)	-	-
	hM3D ChaCha (sgHBG, - CNO)	hM3D ChaCha (sgHBG, + CNO)	4.6	**0.0003**
S8a	dCas9 VPR (sgGal4/sgGal4)	dCas9 VPR (sgIL2/sgGal4) For IL2 measurements	3.3	**<0.0001**
	dCas9 VPR (sgGal4/sgGal4)	dCas9 VPR (sgGal4/sgIFNG) For IL2 measurements	b.d.l.*	-
	dCas9 VPR (sgGal4/sgGal4)	dCas9 VPR (sgIL2/sgIFNG) For IL2 measurements	3.27	**<0.0001**
	dCas9 VPR (sgGal4/sgGal4)	dCas9 VPR (sgIL2/sgGal4) For IFNG measurements	b.d.l.*	-
	dCas9 VPR (sgGal4/sgGal4)	dCas9 VPR (sgGal4/sgIFNG) For IFNG measurements	20.7	**0.0018**
	dCas9 VPR (sgGal4/sgGal4)	dCas9 VPR (sgIL2/sgIFNG) For IFNG measurements	25	**0.0014**
S8b	hM3D ChaCha (sgGal4/sgGal4, - CNO)	hM3D ChaCha (sgGal4/sgGal4, + CNO) For IL2 Measurements	b.d.l.*	-
	hM3D ChaCha (sgIL2/sgGal4, - CNO)	hM3D ChaCha (sgIL2/sgGal4, -+CNO) For IL2 Measurements	2.8	**<0.0001**
	hM3D ChaCha (sgGal4/sgIFNG, - CNO)	hM3D ChaCha (sgGal4/sgIFNG, + CNO) For IL2 Measurements	b.d.l.*	-
	hM3D ChaCha (sgIL2/sgIFNG, - CNO)	hM3D ChaCha (sgIL2/sgIFNG, + CNO) For IL2 Measurements	3.3	**<0.0001**
	hM3D ChaCha (sgGal4/sgGal4, - CNO)	hM3D ChaCha (sgGal4/sgGal4, + CNO) For IFNG Measurements	b.d.l.*	-
	hM3D ChaCha (sgIL2/sgGal4, - CNO)	hM3D ChaCha (sgIL2/sgGal4, -+CNO) For IFNG Measurements	b.d.l.*	-
	hM3D ChaCha (sgGal4/sgIFNG, - CNO)	hM3D ChaCha (sgGal4/sgIFNG, + CNO) For IFNG Measurements	11	**0.0006**

	hM3D ChaCha<br>(sgIL2/sgIFNG, - CNO)	hM3D ChaCha<br>(sgIL2/sgIFNG, + CNO)<br>For IFNG Measurements	13	**0.0002**
S9	dCas9-VPR (N)	dCas9-VPR (T)	33x	**0.0004**
	CXCR4 ChaCha (- SDF1)<br>Variant A	CXCR4 ChaCha (+ SDF1)<br>Variant A	2.0	**0.0018**
	CXCR4 ChaCha (- SDF1)<br>Variant L	CXCR4 ChaCha (+ SDF1)<br>Variant L	3.3	**0.0005**
	CXCR4 ChaCha (- SDF1)<br>Variant M	CXCR4 ChaCha (+ SDF1)<br>Variant M	5.1	**0.0003**
	NMBR ChaCha (- NMB)<br>Variant A	NMBR ChaCha (+ NMB)<br>Variant A	8.7	**0.0069**
	NMBR ChaCha (- NMB)<br>Variant L	NMBR ChaCha (+ NMB)<br>Variant L	1.2	0.2908
	NMBR ChaCha (- NMB)<br>Variant M	NMBR ChaCha (+ NMB)<br>Variant M	1.1	0.7922
	LPAR ChaCha (- NMB)<br>Variant A	LPAR ChaCha (+ NMB)<br>Variant A	2.0	0.1212
	LPAR ChaCha (- NMB)<br>Variant L	LPAR ChaCha (+ NMB)<br>Variant L	2.8	**0.0245**
	LPAR ChaCha (- NMB)<br>Variant M	LPAR ChaCha (+ NMB)<br>Variant M	1.2	0.2483
S11	dCas9-VPR (sgGal4)	dCas9-VPR (sgTet)	39	**<0.0001**
	hM3D ChaCha (- CNO)	hM3D ChaCha (+ CNO)	2.16	**<0.0001**
	hM3D ChaCha ΔTEVp (-CNO)	hM3D ChaCha ΔTEVp (+CNO)	0.74	**0.0197**

*For ELISA values below the detection limit of 4 pg/mL, fold changes are estimates compared to that limit; b.d.l., below detectable limit. For sgRNA screening, where only one experiment with technical replicates were performed, only fold change is reported.
