# Supplementary material for: Engineering cell sensing and responses using a GPCR-coupled CRISPR-Cas system
Source: Nat Commun. 2017 Dec 20;8:2212. doi: 10.1038/s41467-017-02075-1 (PMC5738360; doi:10.1038/s41467-017-02075-1)
Supplement: Supplementary file 2 — Description of Additional Supplementary Files [file 41467_2017_2075_MOESM2_ESM.pdf]

Description of Additional Supplementary Files

File Name: Supplementary Data 1
Description: ARRB2-TCS-dCas9-mCherry expression values, related to Fig. 1c.

File Name: Supplementary Data 2
Description: GFP expression values, related to Fig. 1c.

File Name: Supplementary Data 3
Description: dCas9-mCherry expression values, related to Supplementary Fig. 2c.

File Name: Supplementary Data 4
Description: GFP expression values, related to Supplementary Fig. 2c.

File Name: Supplementary Data 5
Description: R script used to generate Fig. 1c and Supplementary Fig. 2c.

File Name: Supplementary Movie 1
Description: Representative time-lapse movie of hM3D-CRISPR ChaCha in HEK293T-GFP reporter cells. Cells were imaged immediately after ±CNO treatment (20 µM) for for 48 h. Upon CNO ligand treatment, hM3D-V2-TEVp-p2A-BFP (not shown) is expected to interact and cleave ARRB2-TCS-dCas9-VPR-mCherry (middle panel). Released dCas9-VPR-mCherry then activates expression of GFP (right panel), which was observed as early as 12 h after CNO treatment. Image frames were taken every 0.5 h. Movie rendered at 7 frames per second. PhC, phase contrast. Scale bar, 50 µm.

File Name: Supplementary Movie 2
Description: Additional time-lapse fields of view of ARRB2-TCS-dCas9-VPRmCherry in HEK293T-GFP reporter cells. Note that nuclear exclusion of mCherry from most cells in these images. Cells were imaged immediately after ±CNO treatment (20 µM) for 48 h. Image frames were taken every 0.5 h. Movie rendered at 7 frames per second. Scale bar, 50 µm.

File Name: Supplementary Movie 3
Description: Additional time-lapse fields of view of GFP reporter activation by hM3DCRISPR ChaCha in HEK293T-GFP reporter cells. Cells were imaged immediately after ±CNO treatment (20 µM) for 48 h. Image frames were taken every 0.5 h. Movie rendered at 7 frames per second. Scale bar, 50 µm.
